# Activity-Regulated Cytoskeleton-Associated Protein Controls AMPAR Endocytosis through a Direct Interaction with Clathrin-Adaptor Protein 2^1^,^2^,^3^

**DOI:** 10.1523/ENEURO.0144-15.2016

**Published:** 2016-05-24

**Authors:** Luis L. P. DaSilva, Mark J. Wall, Luciana P. de Almeida, Sandrine C. Wauters, Yunan C. Januário, Jürgen Müller, Sonia A. L. Corrêa

**Affiliations:** 1 Ribeirão Preto Medical School, University of São Paulo, Ribeirão Preto, São Paulo, 14049-900 Brazil; 2 School of Life Sciences, University of Warwick, Coventry, CV4 7AL United Kingdom; 3 Warwick Medical School, University of Warwick, Coventry, CV4 7AL United Kingdom; 4 Bradford School of Pharmacy, Faculty of Life Sciences, University of Bradford, Bradford BD7 1DP, United Kingdom; 5 Aston Medical Research Institute, Aston Medical School, Aston University, Birmingham B4 7ET, United Kingdom

**Keywords:** : adaptor protein 2, AMPAR endocytosis, clathrin-mediated endocytosis, hippocampus, neuronal excitability, synaptic transmission

## Abstract

The activity-regulated cytoskeleton-associated (Arc) protein controls synaptic strength by facilitating AMPA receptor (AMPAR) endocytosis. Here we demonstrate that Arc targets AMPAR to be internalized through a direct interaction with the clathrin-adaptor protein 2 (AP-2). We show that Arc overexpression in dissociated hippocampal neurons obtained from C57BL/6 mouse reduces the density of AMPAR GluA1 subunits at the cell surface and reduces the amplitude and rectification of AMPAR-mediated miniature-EPSCs (mEPSCs). Mutations of Arc, that prevent the AP-2 interaction reduce Arc-mediated endocytosis of GluA1 and abolish the reduction in AMPAR-mediated mEPSC amplitude and rectification. Depletion of the AP-2 subunit µ2 blocks the Arc-mediated reduction in mEPSC amplitude, an effect that is restored by reintroducing µ2. The Arc–AP-2 interaction plays an important role in homeostatic synaptic scaling as the Arc-dependent decrease in mEPSC amplitude, induced by a chronic increase in neuronal activity, is inhibited by AP-2 depletion. These data provide a mechanism to explain how activity-dependent expression of Arc decisively controls the fate of AMPAR at the cell surface and modulates synaptic strength, via the direct interaction with the endocytic clathrin adaptor AP-2.

## Significance Statement

The direct binding of Arc to the clathrin-adaptor protein 2 complex discovered in this study provides the crucial mechanistic link between the activity-dependent expression of Arc and the targeting of specific synaptic AMPA receptors for endocytosis. The interaction between Arc and AP-2 is crucial for many forms of synaptic plasticity and may provide a novel target for therapeutic intervention.

## Introduction

Activity-dependent long-lasting alterations in glutamatergic synaptic strength are the molecular substrate thought to underlie learning and memory. The establishment and maintenance of changes in synaptic strength is dependent on trafficking of AMPA receptors (AMPAR) at the postsynaptic membrane (Ehlers, 2000; Newpher and Ehlers, 2008), together with changes in protein synthesis (Buffington et al., 2014). In recent years, several neuron specific immediate early genes (IEGs) that are rapidly induced in response to neuronal activity have been described (Flavell and Greenberg, 2008), including activity-regulated cytoskeleton-associated (Arc) protein, also named activity-regulated gene of 3.1 kb (Arg3.1). Following neuronal activation, Arc mRNA is rapidly trafficked to postsynaptic dendritic sites and locally translated (Lyford et al., 1995; Steward et al., 1998). A rapid increase in Arc protein expression regulates synaptic strength, mainly by enhancing the endocytosis of AMPAR at postsynaptic sites (Rial Verde et al., 2006; Shepherd et al., 2006; Waung et al., 2008; Mabb et al., 2014). A number of studies have shown that Arc regulates several forms of synaptic plasticity, including homeostatic scaling (Shepherd et al., 2006; Corrêa et al., 2012; Mabb et al., 2014) and metabotropic glutamate receptor-dependent long-term depression (Waung et al., 2008; Jakkamsetti et al., 2013; Mabb et al., 2014). Arc is also required for inverse synaptic tagging. In this process, strong neuronal stimulation induces Arc expression, which binds to inactive CaMKIIβ (Okuno et al., 2012). The Arc/CaMKIIβ complex then operates as a sensor to identify and induce endocytosis of AMPAR at weaker synapses thus increasing the difference between activated and non-activated synapses. Together, these findings demonstrate a pivotal role for Arc in regulating synapse strength after neuronal activation.

The clathrin-mediated endocytic (CME) pathway has been the subject of intensive studies in the past decades. Therefore, the molecular machinery involved in the sequential events linking the selection of the endocytic cargo and assembly of the clathrin scaffold leading to membrane bending and scission of the newly formed clathrin-coated vesicles has been precisely described (Saheki and De Camilli, 2012; Canagarajah et al., 2013; Kirchhausen et al., 2014). The clathrin-adaptor protein 2 (AP-2), which is a heterotetramer composed of two large (α/β2) and two small (μ2/σ2) subunits, plays an essential role in the formation of endocytic clathrin-coated vesicles (CCV). To initiate the clathrin-coat assembly the AP-2 complex first binds to the transmembrane cargo that is to be internalized and subsequently binds and connects clathrin to the plasma membrane (Saheki and De Camilli, 2012; Traub and Bonifacino, 2013; Kirchhausen et al., 2014). The sequential events observed during clathrin-mediated endocytosis are conserved across different eukaryotic cell types including neurons (Saheki and De Camilli, 2012). In hippocampal neurons, the cytosolic tail of the AMPAR subunit 2 (GluA2) directly binds to AP-2 (Kastning et al., 2007) and disruption of the AMPAR–AP-2 interaction compromises the Arc-mediated facilitation of AMPAR endocytosis (Rial Verde et al., 2006).

Here we show that Arc directly binds to AP-2 and that this interaction is required for Arc-mediated endocytosis of GluA1 subunits and consequent changes in synaptic transmission. Under basal conditions, overexpression of Arc-wild-type (Arc-WT) reduces the amplitude and rectification of AMPAR-mediated miniature EPSCs (mEPSCs), whereas Arc proteins bearing mutations in the AP-2 binding site, have little or no effect. Furthermore, depletion of AP-2 blocks the Arc-mediated reduction in mEPSC amplitude, an effect that is rescued when AP-2 expression is restored. The interaction between Arc and AP-2 is also important in homeostatic synaptic scaling, as depletion of AP-2 significantly reduces the Arc-dependent decrease in AMPAR mEPSC amplitude induced by increased neuronal activity. The discovery that the direct interaction between Arc and AP-2 facilitates rapid and sustained AMPAR endocytosis provides the mechanistic link by which constitutive endocytosis can be regulated by changes in activity in neurons. These findings further consolidate the strategic role of Arc in facilitating activity-dependent endocytosis of AMPAR in synaptic plasticity.

## Materials and Methods

Animals used in this study were treated in accordance with UK Animal (Scientific Procedures) Act 1986 legislation and under the appropriate national and local ethical approval. Sample size was calculated using variance from previous experiments to indicate power, with statistical significance set at 95%. Replication values are incorporated in the figures, where appropriate.

### Immunoprecipitation and immunoblot analysis

To identify new proteins that interact with endogenous Arc/Arg3.1 proteins hippocampi from 10-week-old male C57BL/6 mice were used. To extract the hippocampi, animals were deeply anaesthetized and the brains were rapidly removed and placed in ice-cold artificial CSF consisting of the following (mm): 124 NaCl, 3 KCl, 26 NaHCO_3_, 1.25 NaH_2_PO_4_, 2 CaCl_2_, 1 MgSO_4_, and 10 d-glucose (bubbled with 95% O_2_ and 5% CO_2_). Hippocampi were then isolated from the surrounding tissue and cut into small pieces using a dissecting microscope (Leica LED 1000). The tissue was then homogenized in Eppendorf Scientific tubes with a pellet pestle in ice-cold solution composed of: 10 mm HEPES, 0.32 m sucrose, and protease inhibitor cocktail (Roche) and rotated for 1 h at 4°C. Homogenate was centrifuged at 13,000 × *g* for 15 min, the supernatant collected and protein levels determined (BCA protein assay kit, Thermo Scientific). Five-hundred micrograms of protein, making 500 µl of final volume, was incubated with 1 µg of rabbit polyclonal anti-Arc antibody (Synaptic Systems, 156-003) and 15 µl of prewashed protein G agarose beads (Upstate-Millipore, 16-266) and rotated for 3 h at 4°C. As a negative control, 500 µg of protein was incubated with 15 µl with protein G agarose beads only. Arc-immunoprecipitation (IP) and negative control samples were centrifuged at 7000 × *g* for 30 s to precipitate the beads. The supernatant was removed and the beads washed three times with lysis buffer containing 1 mm EDTA, 1 m Tris-HCl, pH 7.5, 1% Triton X-100, 1 mm sodium orthovanadate, 50 mm sodium fluoride, sodium pyrophosphate, 0.27 m sucrose, 20% NaN_3_, and protease inhibitor cocktail (Roche). Proteins were eluted from the beads with 20 µl of 5× loading buffer, and the total amount of the eluted protein from the beads were loaded into a 10% SDS-PAGE gels and separated for 1.5 cm using electrophoresis system.

To further confirm the endogenous interaction between Arc and AP-2 in the hippocampus, we used the co-IP experimental conditions described above. Eluted IP proteins, as well as inputs, were separated in 10% SDS-PAGE gels, transferred into membrane using electrophoresis system, and blots were incubated overnight with primary antibodies: rabbit anti-Arc/Arg3.1 (1:1000 dilution), mouse anti-α-adaptin1/2 (1:1000 dilution, sc-17771), and goat anti-clathrin HC (1:1000 dilution, sc-6579). Normal Rabbit IgG (1:1000; R&D Systems, AB-105-C) was used as negative control for the IP experiments. Appropriate secondary antibodies were used to detect proteins levels.

### Proteomics and MS analysis

Each gel lane (Arc-IP and control) were cut in small pieces and subjected to in-gel tryptic digestion using a ProGest automated digestion unit (Digilab). The resulting peptides were fractionated using a Dionex Ultimate 3000 nanoHPLC system. Briefly, peptides in 1% (v/v) formic acid were injected onto an Acclaim PepMap C18 nano-trap column (Dionex). After washing with 0.5% (v/v) acetonitrile 0.1% (v/v) formic acid peptides were resolved on a 250 mm × 75 μm Acclaim PepMap C18 reverse phase analytical column (Dionex) over a 120 min organic gradient with a flow rate of 300 nl min^−^1. Peptides were ionized by nano-electrospray ionization at 2.3 kV using a stainless steel emitter with an internal diameter of 30 μm (Proxeon). Tandem mass spectrometry analysis was carried out on a LTQ-Orbitrap Velos mass spectrometer (Thermo Scientific). The Orbitrap was set to analyze the survey scans at 60,000 resolution and the top 20 ions in each duty cycle selected for MS/MS in the LTQ linear ion trap. Data were acquired using the Xcalibar v2.1 software (Thermo Scientific). The raw data files were processed and quantified using Proteome Discoverer software v1.2 (Thermo Scientific) with searches performed against the UniProt rat database by using the SEQUEST algorithm with the following criteria; peptide tolerance = 10 ppm, trypsin as the enzyme, carbamidomethylation of cysteine as a fixed modification and oxidation of methionine as a variable modification. The reverse database search option was enabled and all data were filtered to satisfy false discovery rate of <5%. Only hits from the Arc-co-IPs were considered for further characterization. The proteomics experiments were repeated twice.

### Hippocampal cell culture and transfection

Hippocampal neuronal cultures were prepared from either male or female postnatal day 0 pups from C57BL/6 wild-type mice as described previously (Canal et al., 2011). Briefly, hippocampi were extracted from the brain at 4°C, subject to digestion with trypsin (Sigma-Aldrich), and mechanically dissociated with DNAse (Sigma-Aldrich). Cells were plated onto 22 mm glass coverslips coated with poly-l-lysine hydrobromide (0.5 mg/ml, Sigma-Aldrich). The plating medium consisted of Neurobasal-A medium (Invitrogen) supplemented with Gentamycin (ForMedium), l-Glutamine (ForMedium), 2% B27 (Invitrogen), and 5% horse serum (Invitrogen). The following day, the plating medium was changed for horse serum-free feeding medium. Cultures were maintained at 37°C and 5% CO_2_ in a humidified incubator. For immunocytochemistry and patch-clamp recordings, hippocampal cultured neurons were used at 14–16 days *in vitro* (DIV) and transfection were performed using Lipofectamine 2000 (Life Technologies). For the patch-clamp recordings, cells expressing Arc cDNAs were used 15–22 h after transfection and cells expressing shRNAs were transfected at 6–7 DIV and recorded at 14–16 DIV.

### Cell lineages culture and transfection

Human neuroglioma 4 (H4) cells obtained from the American Type Culture Collection were cultured in DMEM (Life Technologies), supplemented with 100 U of penicillin/ml, 0.1 mg of streptomycin/ml, and 10% (vol/vol) fetal bovine serum, and then transiently transfected using Lipofectamine 2000 (Life Technologies). Neuroblastoma × Spinal Cord (NSC) hybrid mouse cell lines (Cashman et al., 1992) cultured in supplemented DMEM were transfect with negative control (n.c.) shRNA, µ2-shRNA_2,_ µ2-shRNA_3_ constructs using calcium phosphate as previously described (Canal et al., 2011). After 72–96 h of transfection, cells were washed, lysed in the presence of protease inhibitor cocktail (Roche), and 10 µg of protein were loaded onto a 10% acrylamide gel. Proteins were separated using an SDS-PAGE system and transferred onto Hydrobond-ECL membrane (GE Healthcare). Membranes were incubated overnight with primary specific mouse anti-AP-50 µ2 subunit antibody (1:500 dilution; BD 610350), and GAPDH (1:1000 dilution; Abcam ab8245 for Fig. 6) or affinity purified rabbit polyclonal anti-GAPDH antibody (1:1000 dilution; Sigma-Aldrich G9545, for Fig. 3). The membranes were incubated with appropriate HRP-linked secondary antibodies anti-Mouse IgG (Cell Signaling Technologies, 7076), anti-Mouse IgG (NA931V, GE Healthcare) or anti-rabbit IgG (NA934V, GE Healthcare) incubated for 1 h at room temperature and blots developed using ECL reagents.

### Recombinant DNA constructs

Full-length mouse Arc cDNA (NM_018790.3) in pCMV-SPORT7 vector was purchased from Open Biosystems and used as a template to generate the Arc constructs. The pGFP-Arc plasmid was generated by cloning the Arc full-length sequence as an EcoRI/SalI fragment into the pEGFP-C2 vector (Clontech). Site-directed mutagenesis (QuickChange II kit, Qiagen) was used to mutate the tryptophan 197 to alanine in the pEGFP-Arc_(WT)_ construct. To generate constructs encoding Arc195-199A, a synthetic cDNA sequence was obtained from GenScript, encoding the mouse Arc residues 1 to 700, in which codons to residues 195–199 (residues QSWGP) of the original Arc sequence were replaced by codons to alanine (QSWGP/AAAAA). The Arc195-199A mutant sequence was then used to replace the corresponding sequence in pGFP-ArcWT, using EcoRI and a naturally occurring BglII (nt 647-652) restriction sites. This generated the pGFP-Arc_(W197A)_ and the pGFP-Arc_(195-199A)_ plasmids, respectively. The plasmids encoding untagged Arc and Arc fused to mCherry (WT and mutants) were obtained by inserting the Arc cDNAs from pEGFP plasmids as EcoRI/SalI fragments into the pCIneo (Promega) or the pmCherry-C2 vectors (Clontech), respectively. To express Arc and Arc mutants in *Escherichia coli* the full-length Arc (WT), Arc 1-194 and Arc 1-199 sequences were amplified by PCR with specific primers and cloned into the pET28a vector using EcoRI and SalI restriction sites. The resulting plasmids encode Arc fused to a hexahistidine tag at the N-terminus. To express GST-Arc_(WT)_, GST-Arc_(195-199A)_, and GST-Arc_(W197A)_ fusion proteins in *E. coli*, the Arc coding sequences in pEGFP-C2 were subcloned into pGEX5.1 (GE Healthcare) as EcoRI/SalI inserts. The pcDNA3.1-µ2-mCherry vector was used to express µ2-adaptin in rescue experiments. This construct was generated using a two-step cloning strategy. Firstly, cDNA encoding mouse µ2 was amplified from pGADT7-µ2 (Guo et al., 2013) and used to replace the Leucine Zipper (LZ) sequence, in a pcDNA3.1-based plasmid consisting of a LZ sequence followed by a linker and the C-terminal (VC: 159-239) fragment of Venus YFP, provided by Dr Stephen Michnick (MacDonald et al., 2006). This construct was subsequently used to replace the VC sequence by the mCherry sequence, thus generating pcDNA3.1-µ2-mCherry. To obtain the GFP-tagged Dynamin2 (WT) construct, the open reading frame of dynamin 2 was cloned into pEGFP-N1 as a HindIII and EcoRI insert. The pEGFP-C3 based plasmid encoding GFP-Triad3A was previously described (Mabb et al., 2014). All open reading frames were verified by nucleotide sequence analysis.

### Recombinant protein expression and GST pull-down assays

The four subunits of rat AP-2 complex comprising residues 1–621 from αC adaptin (α-trunk) fused to glutathione-S-transferase (GST) at the N-terminus, residues 1–591 from β2 adaptin fused to a hexahistidine tag at the C-terminus, and the full-length μ2 and σ2 adaptin; (hereafter referred to as AP2 core) were coexpressed in *E. coli* BL21 Rosetta (DE3) cells from a pST39 vector (Sheffield et al., 1999) under the control of T7 promoter with each gene having its own ribosome-binding site (Chaudhuri et al., 2007; Chaudhuri et al., 2009). For GST-AP-2 core expression, bacteria were grown at 37°C to an optical density at 600 nm of 0.8. Then cultures were shifted to 18˚C and the expression was induced with 0.2 mm IPTG (isopropyl-β-d-thiogalactopyranoside) for 12 h. The cell pellet was resuspended in ice-cold lysis buffer (50 mm Tris-HCl, pH 7.4, 150 mm NaCl, 10% glycerol, 2 mm EDTA, 10 mm DTT), supplemented with 500 µg/ml lysozyme and 1 mm 4-(2-aminoethyl) benzenesulfonyl fluoride hydrochloride and disrupted by sonication. Insoluble material was removed by centrifugation and the AP-2 core in the supernatant was purified using a His-trap column (GE Healthcare). Briefly, the AP-2 core complex was bound to the His-trap column via the 6xHis-β2 subunit, repeatedly washed with Tris-buffer solution (TBS) composed of 50 mm Tris-HCl, pH 7.4, 500 mm NaCl supplemented with 30 mm of imidazol (Sigma-Aldrich) and eluted with TBS with 0.25 m of imidazol. Recombinant GST (pGEX plasmid), GST-Arc_(WT)_, GST-Arc_(195-199A)_, GST-Arc_(W197A)_, and 6XHis-Arc (wild-type and truncated) were also expressed in *E. coli* BL21 Rosetta (DE3) cells at 30°C with 0.5 mm IPTG. The pellet was resuspended in ice-cold lysis buffer, sonicated and after centrifugation, and supernatant containing the soluble proteins was used for pull-down assays.

Recombinant GST-AP-2 core or GST alone was immobilized onto glutathione-sepharose beads (GE Healthcare) overnight at 4°C. Beads were washed with ice-cold TBS containing 5% of Triton X-100 (Sigma-Aldrich) and incubated with either His-trap column purified 6xHis-Arc or total cell lysates of *E. coli* expressing 6x-His-Arc proteins for 3 h. After four washes with ice-cold TBS plus 5% of Triton X-100 the beads were resuspended in sample buffer (SDS 4%, Tris-HCl 160 mm, pH 6.8, glycerol 20%, DTT 100 mm, and bromophenol blue 0.005%), boiled, and proteins were separated by SDS-PAGE and transferred onto a nitrocellulose membrane (GE Healthcare), which were then blocked for 1 h with PBS, 0.1% Tween 20, and 5% milk powder. Primary mouse monoclonal anti-His tag antibody (1:1000 dilution, Sigma-Aldrich H1029) were added in PBS, 1% BSA for 1 h. After three washes with PBS-T, the membranes were incubated with HRP-conjugated secondary antibody for 1 h and washed again.

Recombinant GST, GST-Arc_(WT)_, GST-Arc_(W197A)_, and GST-Arc_(195-199A)_ were immobilized onto glutathione-sepharose beads overnight at 4°C. Beads were incubated with either total brain tissue lysate, obtained as described earlier for hippocampi lysate, or total lysates of HEK293 cells expressing either dynamin 2-GFP or GFP-Triad3A for 1 h at 4°C on ice. The beads were centrifuged at 100 × g, washed three times with lysis buffer, supplemented with 1% (v/v) Triton X-100, and subsequently resuspended in SDS-PAGE sample buffer. Beads were boiled, and proteins were resolved by SDS-PAGE and analyzed by immunoblot as described above using mouse monoclonal anti-AP-50 µ2 subunit (1:500 dilution; BD 611350), anti-α-adaptin1/2 (1:1000 dilution, sc-17771), and rabbit polyclonal anti-GFP antibodies. Proteins were detected using ECL reagents.

### Immunocytochemistry

H4 neuroglioma cells (ATCC) were transfected with plasmids encoding a myc-tag at the N-terminus of GluA1 (Leuschner and Hoch, 1999) together with plasmids encoding either mCherry, mCherry-Arc-WT, or mCherry-Arc_(W197A)_. Twenty hours after transfection, cells were fixed using 4% paraformaldehyde, pH 7.4, in 0.1 m PBS for 15 min at room temperature and incubated for 30 min at 37°C in blocking solution (0.2% pork skin gelatin) in PBS. Cells were then incubated with hybridoma culture supernatant (9E10) containing mouse monoclonal anti-myc antibody (at 1:10 dilution). Cells were washed with PBS and incubated with AlexaFluor 488 anti-mouse IgG (1:1000; Life Technologies) diluted in blocking solution. Cells were then permeabilized for 10 min with 0.1% Triton X-100 in PBS and incubated again with rabbit polyclonal anti-myc antibody (a gift from R. Hegde, MRC, LMB, Cambridge, UK) for 30 min at 37°C in blocking solution. This was followed by incubation with AlexaFluor 647 anti-rabbit IgG (1:1000; Life Technologies) diluted in blocking solution. Coverslips were mounted on glass slides, and cells were imaged using a Zeiss LSM 780 confocal microscope.

### Biotinylation assays

To analyze the amount of surface and intracellular GluA1 and GluA2 proteins H4 neuroglioma cells were transfected and subject to a biotinylation protocol previously described (Eales et al., 2014). Briefly, the same amount of H4 cells were seeded in each well of 6-well dishes (3 × 10^5^ cells/well) and then transfected with 2 μg of plasmids encoding N-terminus myc-tagged GluA1 or GluA2 (Leuschner and Hoch, 1999) in combination with 2 μg of pCIneo, pCIneo-Arc_(WT)_, or pCIneo-Arc_(W197A)_ using Lipofectamine 2000. After 24 h, the cells were washed and incubated with 1 ml of 0.25 mg/ml of EZ-Link Sulfo-NHS-SS-Biotin (Thermo Scientific) in ice-cold PBS for 15 min at 4°C. The cells were washed twice with ice-cold PBS, with 3 ml of NH4Cl 50 mM for 5 min (4°C on a shaker), and then once more with PBS. After washing, cells were lysed with 100 µl of lysis buffer (described above) containing protease inhibitors, rotated for 1 h at 4°C, centrifuged at 20,000 × *g* for 10 min at 4°C and the supernatants collected. The protein concentration was assayed using the BioRad Protein Assay Reagent and equal amounts were incubated with prewashed 30 μl of NeutrAvidin Ultra-link Resin (Life Technologies) for 3 h on a wheel at 4°C. The beads were washed three times with lysis buffer, and the proteins eluted from the beads using 20 µl of 5× loading buffer. Proteins were loaded on an 8% SDS-PAGE gel. The input represents 1% of the total protein incubated with the beads. The Western blot was performed as described above.

### Lentiviruses production

A lentiviral transduction system was used to achieve efficient delivery of specific microRNA-adapted shRNA sequences into neurons. Double-stranded oligonucleotides encoding shRNAs targeting the mouse µ2 subunit (shRNA1: tgctgtgaattgccctccatatggttgttttggccactgactgacaaccatatagggcaattca/cctgtgaattgccctatatggttgtcagtcagtggccaaaacaaccatatggagggcaattcac; shRNA2: tgctgcatattggtactctattgcctgttttggccactgactgacaggcaatagtaccaatatg/cctgcatattggtagtattgcctgtcagtcagtggccaaaacaggcaatagagtaccaatatgc; shRNA3: tgctgatctgcaggacattgcttcacgttttggccactgactgacgtgaagcagtcctgcagat/cctgatagattcctatcaggctggtcagtcagtggccaaaaccagcctgagttaggaatctatc) were cloned into the linearized pcDNA6.2-GW/EmGFP-miR vector (Invitrogen). The sequences were designed using the “BLOCK-iT RNAi Designer” software from Invitrogen to identify sequences specific for mouse µ2 that are not predicted to knockdown expression of any other genes. In addition, the sequences have 100% homology to the target sequence and result in target cleavage. The vector contains flanking sequences allowing the shRNAs to be expressed and processed analogous to endogenous miRNAs and not shRNAs. This arrangement enables the expression of the shRNA cassette from an RNA polymerase II promoter. In addition, emGFP is expressed iso-cistronically from the same promoter to allow the precise identification of the transduced cells. As a negative control, the plasmid pcDNA6.2-GW/EmGFP-miR-neg control (Invitrogen) was used. This plasmid contains an insert that forms a hairpin structure, which is processed into mature shRNA, but is predicted not to target any known vertebrate gene (gaaatgtactgcgcgtggagacgttttggccactgactgacgtctccacgcagtacattt). The above expression cassettes were transferred into the lentiviral expression vector pLenti6/V5-DEST (Invitrogen) by gateway cloning. Lentiviruses were produced according to the instructions of the manufacturer (Invitrogen; Block-It HiPerform Lentiviral Pol II RNAi Expression system with emGFP; K4934). Lentivirus particles were collected from the culture supernatants, purified, and concentrated by incubation with 8.5% PEG 6000 and 0.4mm NaCl for 1.5 h at 4°C, followed by centrifugation at 7000 × *g* for 10 min (4°C). Pellets were re-dissolved in neurobasal medium.

### Bicuculline incubation

To induce a chronic increase in neuronal activity, hippocampal cultures were incubated with bicuculline (40 µm, Sigma-Aldrich) for 48 h prior to experimental work.

### Electrophysiological recordings and analysis of AMPAR-mediated mEPSCs

mEPSCs were recorded from 15 to 18 DIV cultured pyramidal hippocampal neurons (Mabb et al., 2014). A coverslip was transferred to the recording chamber and perfused at a constant flow rate of (2–3 min^−^1) with a recording solution composed of (mm): 127 NaCl, 1.9 KCl, 1 MgCl_2_, 2 CaCl_2_, 1.3 KH_2_PO_4_, 26 NaHCO_3_, 10 d-glucose, pH 7.4 (when bubbled with 95% O2 and 5% CO_2_, 300 mOsm) at 28–30°C. To isolate AMPA receptor mediated mEPSCs, tetrodotoxin (1 µm, Tocris Bioscience), picrotoxin (50 µm, Sigma-Aldrich) and L-689,560 (5 µm, Tocris Bioscience) were present in the recording solution. Cultured neurons were visualized using IR-DIC optics with an Olympus BX51W1 microscope and Hitachi CCD camera (Scientifica). Whole-cell patch-clamp recordings were made from transfected (identified by fluorescence at 488 nm) and neighboring untransfected pyramidal neurons with patch pipettes (5–8 MΩ) made from thick-walled borosilicate glass (Harvard Apparatus) filled with the following (mm): 135 potassium gluconate, 7 NaCl, 10 HEPES, 0.5 EGTA, 10 phosphocreatine, 2 MgATP, 0.3 NaGTP, pH 7.2, 290 mOsm. Recordings of mEPSCs were obtained at a holding potential of −75 mV using an Axon Multiclamp 700B amplifier (Molecular Devices), filtered at 3 kHz and digitized at 20 kHz (Digidata 1440A, Molecular Devices). For rectification experiments, the intracellular solution contained the following (mm): 135 CsCl, 10 HEPES, 10 EGTA, 2 Mg-ATP, 0.1 spermine, pH 7.2 with tetraethylammonium-0H, 285 mOsm. To calculate the rectification index, mEPSC recordings were made at holding potentials of −60 and +40 mV. Data acquisition was performed using pClamp 10 (Molecular Devices).

Analysis of mEPSCs was performed using MiniAnalysis software (SynaptoSoft). For most experiments, where the holding potential was −75 mV, events were manually analyzed and were accepted if they had an amplitude >6 pA and a faster rise than decay. For the rectification experiments, where the holding potential was −60 and +40 mV and thus mEPSCs had a smaller amplitude, events were accepted if they had an amplitude >3 pA and a waveform with a faster rise than decay. Cumulative probability curves for mEPSC amplitude were constructed from 1000 to 2000 mEPSCs pooled from all recordings, with the same number of mEPSCs (150) measured from each recording (Origin, Microcal). The interval between events was measured using MiniAnalysis software. To measure mEPSC kinetics, mEPSCs within individual recordings were aligned on the half-amplitude of their rise and averaged (50–100 mEPSCs were averaged in each recording). The decay of the mean current from each recording was fitted with a single exponential (maximum likelihood, MiniAnalysis or Microcal Origin). Rise times were measured from mean currents as the time required for the current to rise from 10% to 90% of peak amplitude. The rectification index was calculated for each recording (peak amplitude at +40 mV divided by peak amplitude at −60 mV), and then the mean rectification index was calculated for each experimental condition. For each cell an average of 100–200 mEPSCs were analyzed. Individual mEPSCs were aligned to 50% of the rise, averaged and then the mean amplitude was measured from the peak of this mEPSC waveform. Statistical significance was measured using the Mann–Whitney test. Where possible, comparisons were made between transfected and untransfected neighboring neurons in the same culture. For each experimental condition, cells were recorded and analyzed using hippocampal cultures from 4 to 5 different preparations.

### Statistical analysis

Data were analyzed using Prism (v5.04, GraphPad) and Statistical Package for the Social Sciences 21 (IBM) software. Mann–Whitney *t* tests, Kolmogorov–Smirnov test, one-way ANOVA, and the corresponding *post hoc* tests (Tukey or Dunn’s) were performed as appropriate.

## Results

### Arc interacts with the AP-2 complex in neurons

**Figure 1.**
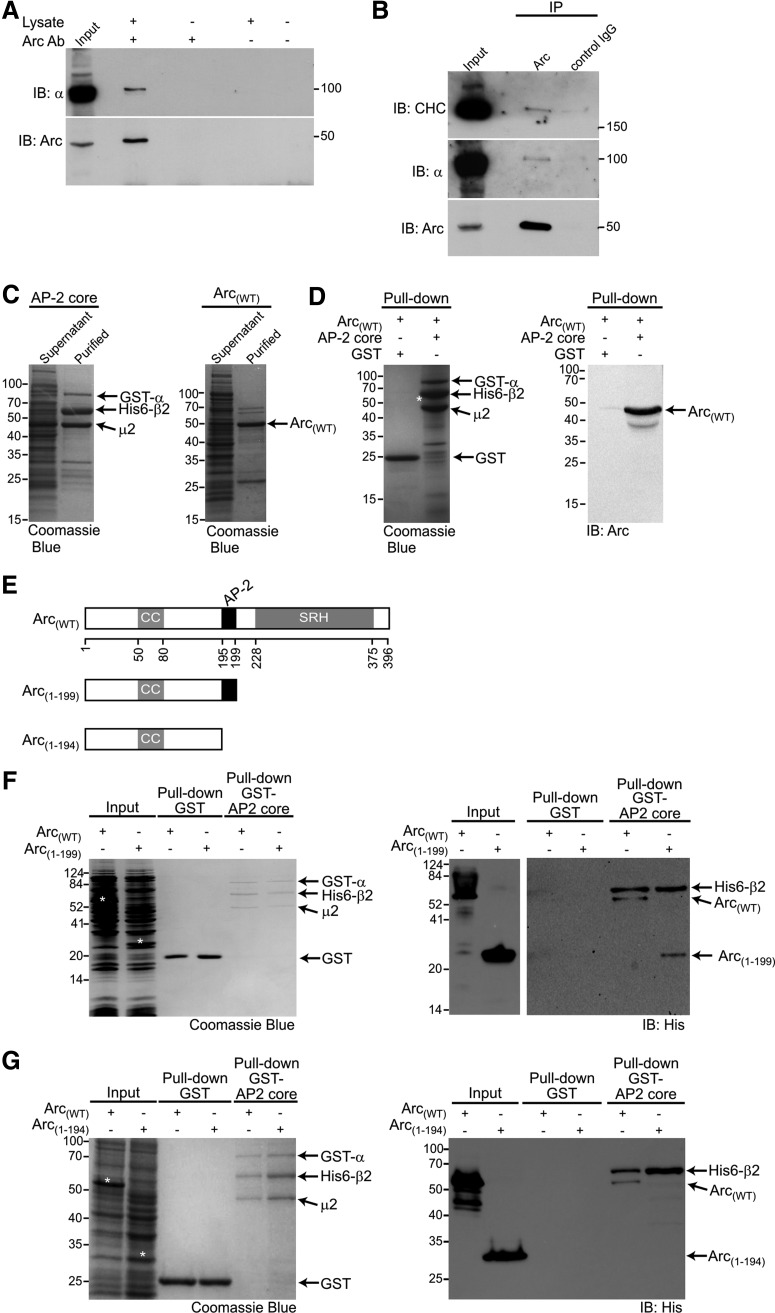
Arc directly interacts with the AP-2 complex. ***A***, Arc coimmunoprecipitates with the α subunit of AP-2. Hippocampal lysate was subjected to IP with an Arc antibody followed by immunoblot (IB) using an anti-α adaptin antibody. Ten percent of the protein lysate used for the IP was loaded in the input lane. ***B***, Arc coimmunoprecipitates with clathrin in hippocampal lysate. Hippocampal lysate was subjected to IP with a rabbit anti-Arc or a normal rabbit anti-IgG control antibodies followed by IB using an anti-α adaptin and anti-clathrin heavy chain antibodies. Ten percent of the protein lysate used for the IP was loaded in the input lane. ***C***, ***D***, Pull-down assay showing the interaction of AP-2 core with mouse Arc_(WT)_. Recombinant affinity purified GST-AP-2 core [GST-tagged α subunit (residues 1–621), 6xHis tagged β2 subunit (residues 1–591), full-length µ2 and σ2 subunits], was immobilized on glutathione beads (***C***, right) and incubated with recombinant affinity purified 6xHis-Arc_(WT)_ (***C***, left). Binding of Arc protein to GST-tagged AP-2 core or GST alone was analyzed by GST pull-down and SDS-PAGE, followed by Coomassie blue staining (***D***, left) or immunoblot using an anti-Arc antibody (***D***, right). ***E***, Schematic representation of the Arc-WT sequence showing the truncated Arc mutants used in this study. The diagram indicates coiled-coil (CC) and spectrin repeat homology (SRH) structure domain of mouse Arc. AP-2 binding site is shown in black. ***f***, Pull-down assay showing the interaction of AP-2 core with mouse Arc_(WT)_ and the Arc_(1-199)_ truncated (deletion of residues 200–396). Recombinant affinity purified AP-2 core, was immobilized on glutathione beads and incubated with lysates of *E. coli* expressing Arc_(WT)_ or Arc_(1-199)_ deletion mutant. Binding of Arc proteins to GST-tagged AP-2 or GST alone was analyzed by GST pull-down and SDS-PAGE, followed by Coomassie blue staining (left) or immunoblot using an anti-His tag antibody (right). Ten percent of the recombinant proteins used for the pull-down were loaded on the input lane (Bands corresponding to Arc proteins are indicated by white asterisks). ***G***, Pull-down assay showing that the Arc residues 195–199 are required for the Arc–AP-2 interaction as truncated Arc_(1-194)_ produced in *E. coli* lost the ability to bind immobilized recombinant GST-tagged AP-2 core.

Arc has been shown to regulate glutamatergic synaptic transmission by dynamically enhancing AMPAR endocytosis in postsynaptic neurons (Shepherd et al., 2006; Mabb et al., 2014). Given the importance of Arc in facilitating AMPAR endocytosis during synaptic transmission, we speculated that it may play a decisive role in selecting the cargo to be internalized. To test whether Arc interacts with proteins of the CME machinery and whether Arc is involved in selecting the cargo to be targeted for endocytosis, we used the specific rabbit anti-Arc antibody to IP endogenous Arc from adult C57BL6/J mice hippocampal lysate combined with mass spectrometric analysis to identify novel Arc binding partners. The control for the IP was obtained by incubating hippocampal lysate protein with the G agarose beads in the absence of Arc antibody. The eluted proteins from both Arc-IP and control-IP samples were subjected to tandem mass spectrometry analysis. We only considered peptides present in the Arc-IPs for further analysis and discarded unspecific peptides present in both Arc- and control-IPs. Using this criteria we identified different subunits of the AP-2 as endogenous binding partners of Arc, including the two α adaptin isoforms: α also known as αA (19 peptides and recovery of 22.83%; NP_031484) and α2, also known as αC (11 peptides and recovery of 12.37%; NP_031485), as well as β2 (11 peptides and recovery of 12.38%; NP_082191) and μ2 (9 peptides and recovery of 20.79%; Q3TWV4). These peptides were found independently in two experimental repeats. We also found clathrin heavy chain (30 peptides and recovery of 20%; NP_001003908), dynamin 1 (10 peptides and recovery of 10.57%; NP_034195), CamKII β subunit (9 peptides and recovery of 20.48%, NP_031621), and PSD95 (2 peptides and recovery 5.77%, NP_031890). Importantly, PSD95, dynamin, and CamKIIβ were previously shown to co-IP with Arc (Lyford et al., 1995; Chowdhury et al., 2006; Okuno et al., 2012). To further confirm that Arc interacts with AP-2 endogenously, we immunoprecipitated Arc protein from hippocampal lysate as previously described and resolved the proteins using SDS-PAGE. Immunoblot analysis confirmed that Arc coimmunoprecipitates with the α subunit of the AP-2 complex (Fig. 1*A*,*B*). We observed that clathrin heavy chain also coimmunoprecipitates with Arc (Fig. 1*B*). This result was expected as clathrin heavy chain is known to interact with AP-2 (ter Haar et al., 2000; Edeling et al., 2006; Knuehl et al., 2006). Together these findings suggest an interaction between Arc and the proteins of the CME machinery that are responsible for selecting the cargo to be internalized. To test whether Arc directly interacts with AP-2, we performed *in vitro* GST pull-down assays using recombinant forms of Arc-WT and AP-2. Previous studies used recombinant AP-2 “core” complexes to demonstrate the direct interaction between AP-2 and the cytosolic tail of transmembrane cargo proteins (Höning et al., 2005) or the HIV-1 accessory protein, Nef (Chaudhuri et al., 2007; Lindwasser et al., 2008; Chaudhuri et al., 2009). Therefore, we produced recombinant Arc-WT fused to a hexahistidine tag and recombinant GST-tagged AP-2 core, comprising the N-terminal “trunk” domains of α and β2 subunits, plus the full-length μ2 and σ2 subunits in *E. coli*. The recombinant AP-2 core complex and Arc proteins were affinity purified (Fig. 1*C*) and used to show that GST-tagged AP-2 core binds mouse Arc-WT, as detected by immunoblot analysis (Fig. 1*D*). We then used the same GST-pull-down approach to map the region of Arc that interacts with AP-2. Our initial experiments demonstrated that a C-terminal fragment of Arc comprising residues 155–396 is sufficient to mediate the interaction with AP-2. We then tested whether Arc mutants bearing cumulative C-terminal deletions of 40 amino acid (aa) aa residues would retain the capacity to bind AP-2. Using this approach, we showed that the Arc C-terminus (residues 200–396; Fig. 1*E*) is not essential for the Arc–AP-2 interaction, as truncated Arc missing these residues (Arc1-199) was still able to interact with AP-2 (Fig. 1*F*). Interestingly, deletion of a further 5 aa residues from the C-terminus of Arc (Arc1-194) was sufficient to prevent AP-2 binding (Fig. 1*G*). Binding of Arc recombinants to GST alone was negligible, thus confirming the specificity of the Arc–AP-2 interactions (Fig. 1*D*,*F*,*G*). Together, these results demonstrated a direct and specific interaction of Arc with the fully assembled AP-2 core complex.

### Conservative tryptophan 197 mediates the Arc–AP-2 interaction

**Figure 2.**
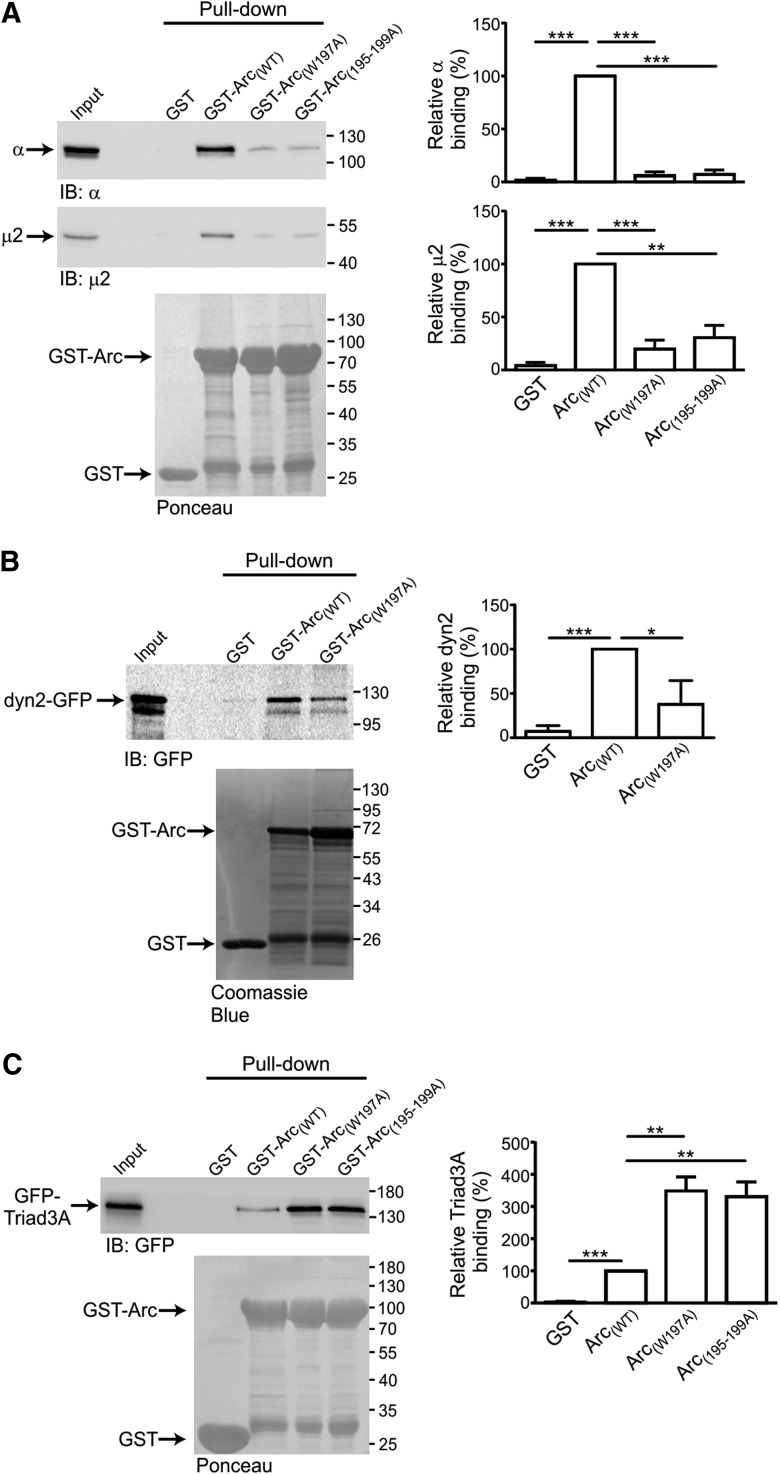
**Identification of Arc motif that binds to AP-2. *A***, Pull-down assay showing that a conserved tryptophan residue at position 197 mediates Arc–AP-2 interaction. Recombinant GST-Arc_(WT)_, GST-Arc_(W197A)_, GST-Arc_(195-199A)_, or GST alone were produced in *E. coli* and immobilized on glutathione beads (bottom) and incubated with total brain tissue lysate. Binding of endogenous µ2 and α-adaptins to GST fusion proteins was analyzed by SDS-PAGE immunoblotting with anti-µ2 (top) or anti-α (middle) antibodies. Bar chart plotting analysis of the relative amount of protein bound to GST and GST-Arc_(W197A)_ and GST-Arc_(195-199A)_ normalized to GST-ArcWT (100%). ***B***, Pull-down assay showing interaction of Arc_(WT)_ and Arc_(W197A)_ with dynamin 2. Recombinant GST-Arc_(WT)_, GST-Arc_(W197A)_, or GST alone were produced in *E. coli*, immobilized on glutathione beads (bottom) and incubated with total lysates of HEK293 cells expressing dynamin 2-GFP. Binding of dynamin 2-GFP to GST fusion proteins was analyzed by SDS-PAGE immunoblotting with anti-GFP antibody (top). Bar chart plotting analysis of the relative amount of dynamin 2 bound on the beads normalized to GST-ArcWT (100%). Ten percent of total protein lysate used to incubate the beads was used as input. ***C***, Pull-down assay showing interaction of Arc_(WT)_, Arc_(W197A)_, and GST-Arc_(195-199A)_ with Triad3A. Recombinant GST proteins produced in *E. coli* and immobilized on glutathione beads (bottom) were incubated with total lysates of HEK293 cells expressing GFP-Triad3A. Binding of GFP-Triad3A to GST fusion proteins was analyzed by SDS-PAGE immunoblotting with anti-GFP antibody (top). Bar chart plotting analysis of the relative amount of Triad3A bound on the beads normalized to GST-ArcWT (100%). Ten percent of total protein lysate used to incubate the beads was used as input. Errors bars represent mean ± SEM (*n* = 3 independent experiments). *p<0.05, **p<0.005, and ***p<0.0005 using unpaired Student´s *t* test.

Our GST pull-down experiments indicate that the Arc _195_QSWGP_199_ amino-acid sequence is required for its interaction with AP-2. Therefore, we reasoned that a single substitution of the highly conserved tryptophan in position 197, may compromise the Arc–AP-2 interaction. To test this, we performed *in vitro* protein-binding experiments using immobilized recombinant GST-Arc_(WT)_, GST-Arc_(195-199A)_, or GST-Arc_(W197A)_ fusion proteins to pull-down the endogenous α or μ2 subunit of AP-2 from total brain tissue lysates. We detected a robust interaction between GST-Arc_(WT)_ and either α or μ2 (Fig. 2*A*; Table 1). However this interaction was dramatically reduced when GST-Arc_(W197A)_, Arc_(195-199A)_, or GST alone were used as bait (Fig. 2*A*), indicating that W197 is crucially involved in the interaction with AP-2. It was previously shown that Arc interacts with dynamin-2 and that an internal deletion of 195–214 aa in Arc disrupt this interaction (Chowdhury et al., 2006). To test the capacity of Arc_(W197A)_ to interact with dynamin, we performed similar in *in vitro* binding analyses using immobilized GST-Arc_(WT)_ or GST-Arc_(W197A)_ to pull-down GFP-dynamin-2 from HEK293 cell lysates. We confirmed that Arc_(WT)_ binds to dynamin, however, there is a significant reduction in the interaction between Arc_(W197A)_ mutant and dynamin (Fig. 2*B*). In contrast, the Arc mutants carrying alanine substitutions in the AP-2 binding motif still interact with the RING domain of the ubiquitin ligase Triad3A/RNF216 (Fig. 2*C*), a protein recently described to interact with Arc (Mabb et al., 2014). Binding of Arc_(W197A)_ and Arc_(195-199A)_ to Triad3A indicates that these alanine mutations do not cause gross conformational changes in Arc which could prevent protein–protein interaction.

**Table 1.** Statistical analyses

**Results**		**Data structure**	**Type of test**	***n* numbers**	**Probability, *p***
(Fig. 2A, top) IB pull-down α	GST-Arc_(WT)_ vs GST	Two-factor, mean	*t* test	3/3	< 0.0001
	GST-Arc_(WT)_ vs GST-Arc_(W197A)_	Two-factor, mean	*t* test	3/3	< 0.0001
GST-Arc_(WT)_ vs GST-Arc_(195-199A)_	Two-factor, mean	*t* test	3/3	< 0.0001	
(Fig. 2A, middle) IB pull-down µ2	GST vs GST-Arc_(WT)_	Two-factor, mean	*t* test	3/3	< 0.0001
	GST-Arc_(WT)_ vs GST-Arc_(W197A)_	Two-factor, mean	*t* test	3/3	0.0007
GST-Arc_(WT)_ vs GST-Arc_(195-199A)_	Two-factor, mean	*t* test	3/3	0.0039	
(Fig. 2B) IB pull-down dyn2-GFP	GST-Arc_(WT)_ vs GST	Two-factor, mean	*t* test	3/3	< 0.0001
GST-Arc_(WT)_ vs GST-Arc_(W197A)_	Two-factor, mean	*t* test	3/3	0.0159	
(Fig. 2C) IB pull-down GFP-Triad3A	GST-Arc_(WT)_ vs GST	Two-factor, mean	*t* test	3/3	< 0.0001
	GST-Arc_(WT)_ vs GST-Arc_(W197A)_	Two-factor, mean	*t* test	3/3	0.0055
GST-Arc_(WT)_ vs GST-Arc_(195-199A)_	Two-factor, mean	*t* test	3/3	0.0055	
(Fig. 3A) IB Surface GluA1	pCIneo vs pArc_(WT)_	Two-factor, mean	ANOVA Tukey’s	3/3	0.1284
pCIneo vs pArc_(W197A)_	Two-factor, mean	ANOVA Tukey’s	4/4	0.5543	
(Fig. 3B) IB Surface GluA2	pCIneo vs pArc_(WT)_	Two-factor, mean	ANOVA Tukey’s	4/4	>0.9999
pCIneo vs pArc_(W197A)_	Two-factor, mean	ANOVA Tukey’s	4/4	0.9637	
(Fig. 3B) IB Surface EGFR	pCIneo vs pArc_(WT)_	Two-factor, mean	ANOVA Tukey’s	4/4	0.6156
pCIneo vs pArc_(W197A)_	Two-factor, mean	ANOVA Tukey’s	4/4	0.7621	
(Fig. 3F) IF Surface GluA1	mCherry vs mCherry-Arc_(WT)_	Two-factor, mean	ANOVA Tukey’s	59/60	<0.0001
mCherry vs mCherry-Arc_(W197A)_	Two-factor, mean	ANOVA Tukey’s	59/42	0.3438	
(Fig. 3G) IF mCherry expression	mCherry vs mCherry-Arc_(WT)_	Two-factor, mean	ANOVA Tukey’s	3/3	0.5625
mCherry vs mCherry-Arc_(W197A)_	Two-factor, mean	ANOVA Tukey’s	3/3	0.9211	
(Fig. 3H) IB Arc expression	mCherry-Arc_(WT)_ vs mCherry- Arc_(W197A)_	Two-factor, mean	ANOVA Tukey’s	3/3	0.6892
mCherry-Arc_(WT)_ vs mCherry- Arc_(195-199A)_	Two-factor, mean	ANOVA Tukey’s	3/3	0.4951	
(Fig. 4) Arc–AP-2 interaction	Arc_(WT)_ vs untransfected amplitude frequency	Two-factor, mean	Mann–Whitney	12/20	0.0002 0.47
	Arc_(W197A)_ vs untransfected amplitude frequency	Two-factor, mean	Mann–Whitney	13/20	0.121 0.98
	Arc_(195-199A)_ vs untransfected amplitude frequency	Two-factor, mean	Mann–Whitney	10/20	0.372 0.18
eGFP vs untransfected amplitude frequency	Two-factor, mean	Mann–Whitney	7/20	0.376 0.39	
(Fig. 5) cDNA constructs and mEPSC kinetics	All constructs vs untransfected rise decay	Two-factor, mean	Mann–Whitney	6/18	>0.05 >0.05
(Fig. 6) AP-2 requirement for Arc mediated changes in synaptic strength	μ2-miRNA_2_ vs untransfected amplitude frequency	Two-factor, mean	Mann–Whitney	9/12	0.07 0.37
	Arc_(WT)_ + μ2-miRNA_2_ vs untransfected amplitude frequency	Two-factor, mean	Mann–Whitney	16/12	0.52 0.63
	Arc_(WT)_ + n.c.miRNA vs untransfected amplitude frequency	Two-factor, mean	Mann–Whitney	7/12	0.001 0.08
	μ2-miRNA_3_ vs untransfected amplitude frequency	Two-factor, mean	Mann–Whitney	10/8	0.68 0.45
Arc_(WT)_ + μ2-miRNA_3_ vs untransfected amplitude frequency	Two-factor, mean	Mann–Whitney	6/8	0.27 0.14	
(Fig. 7) The Arc-AP-2μ interaction is required for Arc-mediated changes in synaptic strength	Arc_(WT)_ +μ2-miRNA_2_+μ2 vs untransfected amplitude frequency	Two-factor, mean	Mann–Whitney	14/14	0.0001 0.37
Arc_(195-199A)_+μ2-miRNA_2_+μ2 vs untransfected amplitude frequency	Two-factor, mean	Mann–Whitney	9/14	0.46 0.64	
(Fig. 8) AP-2 is required for homeostatic scaling	Control vs bicuculline (untransfected) amplitude frequency	Two-factor, mean	Mann–Whitney	10/15	0.0001 0.64
	miRNA_2_ (bicuculline) vs untransfected (bicuculline) amplitude frequency	Two-factor, mean	Mann–Whitney	6/15	0.0001 0.59
n.c.miRNA (bicuculline) vs untransfected amplitude frequency	Two-factor, mean	Mann–Whitney	5/15	0.007 0.29	

### Arc-mediated internalization of GluA1 requires the Arc–AP-2 interaction

**Figure 3.**
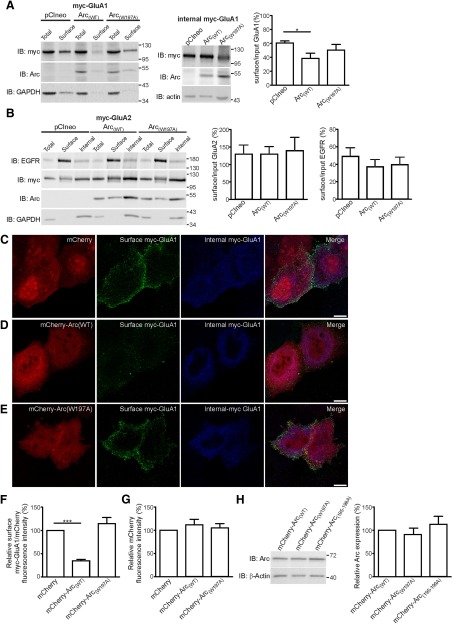
**Arc–AP-2 interaction regulates GluA1 endocytosis. *A***, ***B***, Representative blots showing that Arc_(WT),_ but not the Arc_(W197A)_ mutant, facilitates GluA1, but not GluA2 endocytosis. H4 neuroglioma cells were transfected with plasmids encoding myc-GluA1 (***A***) or myc-GluA2 (***B***) in combination with either: empty pCIneo vector, pCIneo Arc_(WT)_, or pCIneo Arc_(W197A)_. Western blot band densitometry analysis showing that: (***A***) Arc_(WT)_, but not Arc_(W197A)_, promotes a significant reduction in surface expression of GluA1 subunits (control: 60.46 ± 2.97%; Arc_(WT)_: 38.55 ± 7.44%; Arc_(W197A)_: 50.18 ± 8.34%. Error bars represent mean ± SEM (*n* = 3 independent experiments). *p<0.05 using one-way ANOVA followed by Tukeýs post-test. ***B***, Arc_(WT)_ does not promote any changes in surface expression of either GluA2 subunits (control: 132.9 ± 26.66%; Arc_(WT_): 133.2 ± 21,78%; Arc_(W197A)_: 143.9 ± 38.43%) or EGF receptor (control: 51.35 ± 10.43%; Arc_(WT)_: 38.93 ± 8.66%; Arc_(W197A)_: 41.59 ± 8.8%). Error bars represent mean ± SEM (*n* = 4 independent experiments).Ten percent of the protein lysate used for incubate the beads was loaded in the input lane. GAPDH was used as loading controls. ***C***–***F***, ***C***–***G***, H4 cells coexpressing myc-GluA1 with either mCherry construct alone (***C***), mCherry-Arc_(WT)_ (***D***), or mCherry-Arc_(W197A)_ (***E***). Surface myc-GluA1 (non-permeabilized cells, green channel) was identified using mouse anti-myc antibody followed by AlexaFluor 488 secondary antibody and internal myc-GluA1 (permeabilized, magenta channel) was identified by polyclonal rabbit anti-myc antibody followed by AlexaFluor 647 secondary antibody. ***F***, ***G***, The mean florescence intensity (MFI) of AlexaFluor 488 (surface my-GluA1) and mCherry (red channel) were calculated using confocal Z-projection images to quantify the pixel intensity of surface myc-GluA1 and mCherry (total protein expression). ***F***, Ratio of averaged MFI between surface (488)/total protein (mCherry) for control cells (*n*=59 cells) was set to 100% to facilitate comparison. Note that the ratio for surface GluA1 is significantly reduced in cells expressing mCherry-Arc_(WT)_ (34.68 ± 3.13%; *n*= 60 cells) compared with cells expressing mCherry construct alone. Importantly, this reduction is absent in cells expressing the mCherry-Arc_(W197A)_ construct (114.80 ± 13.08%; *n*= 42 cells. ***G***, Bar chart plotting the averaged MFI expression levels of mCherry-Arc_(WT)_ and mCherry-Arc_(W197A)_ compared with mCherry expression. Values are mean ± SEM (*n*=3 independent experiments). *p<0.05, ***p<0.005 using one-way ANOVA followed by Tukeýs post-test. Scale bar, 10 μm. ***H***, Representative blot and bar chart plotting bands densitometry analysis of Arc expression protein in H4 cells transfected with equal amounts of mCherry-Arc_(WT)_, mCherry-Arc_(W197A)_, or mCherry-Arc_(195-199A)_ plasmids. Note the similar levels Arc protein expression between samples. Values are mean ± SEM (*n*=3 independent experiments).

Arc_(WT)_ overexpression in hippocampal neurons reduces surface levels of AMPAR by selectively enhancing endocytosis. We reasoned that Arc-mediated endocytosis of AMPAR may be linked to its ability to interact with the endocytic adaptor AP-2. To test this, we coexpressed myc-GluA1 with either Arc_(WT)_ or the Arc_(W197A)_ mutant in H4 human neuroglioma cells, and performed biotinylation assay to monitor GluA1 and GluA2 surface expression levels. As previously shown in hippocampal neurons (Chowdhury et al., 2006), overexpression of Arc-WT in H4 cells resulted in a significant reduction of myc-GluA1 surface expression levels (Fig. 3*A*; Table 1). Importantly, the reduction in myc-GluA1 surface expression was blocked when Arc_(W197A)_ mutant, that does not bind AP-2, was coexpressed with myc-GluA1 (Fig. 3*A*). Interestingly no changes in GluA2 surface expression were observed when myc-GluA2 construct was coexpressed with either Arc_(WT)_ or the Arc_(W197A)_ mutant (Fig. 3*B*), indicating that the GluA2 subunit is potentially less sensitive to Arc than GluA1 as previously suggested by Chowdhury et al. (2006). To test whether Arc overexpression induces general endocytosis of AP-2/clathrin cargo proteins, we examined the surface levels of EGF receptor (EGFR) in H4 cells expressing either Arc-WT or the Arc_(W197A)_ mutant. As expected, expression of Arc has no significant effect in surface expression of EGFR (Fig. 3*B*). To confirm whether Arc_(W197A)_ mutant had an impact on the Arc-dependent internalization of GluA1, we used the same experimental condition described above to perform immunocytochemistry to label the amount of n terminus-myc-tagged GluA1 expressed at the surface. Confocal microscopy analyses confirmed that Arc_(WT)_ overexpression promotes a significant reduction of the GluA1 expression at the cell surface, an effect that is impaired in cells expressing the Arc_(W197A)_, that cannot bind to AP-2 (Fig. 3*C*–*G*). Together, these results indicate that Arc–AP-2 interaction is required to facilitate AMPAR internalization.

### The Arc–AP-2 interaction regulates AMPAR-mediated synaptic currents

**Figure 4.**
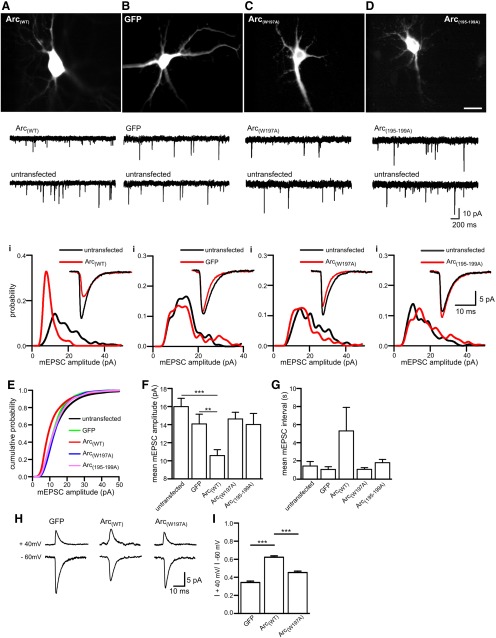
**The Arc–AP-2 interaction regulates AMPAR-mediated synaptic currents. *A***–***D***, Representative live imaging of a dissociated hippocampal neuron overexpressing Arc-GFP-tagged constructs and GFP. ***A***, AMPAR-mediated mEPSC traces from a neuron overexpressing Arc_(WT)_ and an untransfected neighboring neuron. ***Ai***, Amplitude probability distribution for the mEPSCs shown in ***A***. Note the shift to the left and increase in the amplitude of the main peak in the neuron overexpressing Arc_(WT)_, clearly demonstrating the reduction in mEPSC amplitude. Inset, Superimposed average mEPSC waveforms. ***B***, Representative AMPAR-mediated mEPSC traces from a neuron overexpressing GFP and an untransfected neighboring neuron. ***Bi***, Amplitude probability distributions for mEPSCs recorded from the neurons shown in ***B***. Inset, Superimposed average mEPSC waveforms. ***C***, Representative AMPAR-mediated mEPSC traces from a neuron expressing Arc_(W197A)_ and an untransfected neighboring neuron. ***Ci***, Amplitude probability distributions from neurons shown in ***C***. Note that expression of Arc_(W197A)_ produced a smaller reduction in mEPSC amplitude compared with Arc_(WT)_ overexpression. Inset, Superimposed average mEPSC waveforms. ***D***, Representative AMPAR-mediated mEPSC traces from a neuron expressing Arc_(195-199A)_ and an untransfected neighboring neuron. ***Di***, Amplitude probability distributions from neurons shown in ***d***. Note that expression of Arc_(195-199A),_ in which the sequence 195QSWGP199 of Arc was mutated to 195AAAAA199 had little effect on mEPSC amplitude. Inset, Superimposed average mEPSC waveforms. ***E***, Cumulative probability distributions for cells expressing Arc_(WT)_ (12 neurons), Arc_(W197A)_ (13 neurons), Arc_(195-199A)_ (10 neurons), GFP (7 neurons), and for untransfected cells (20 neurons). ***F***, Bar chart plotting mean mEPSC amplitude for the cells in ***E***. Expression of Arc_(WT)_ significantly reduced mEPSC amplitude (mean reduced from 15.99 ± 0.9 pA in untransfected cells to 10.56 ± 0.66 pA, *p*= 0.0002), whereas expression of Arc_(W197A)_ or Arc_(195-199A)_ had no significant effect (14.6 ± 0.74 pA, *p*=0.12 and 14.01 ± 1.2 pA, *p*= 0.37). Expression of eGFP had no significant effect (*p*=0.376) on the mean mEPSC amplitude compared to untransfected cells. ***G***, Bar chart plotting the mean interval between mEPSCs. Expression of Arc_(WT)_ and the Arc mutants had no significant effect on the frequency of mEPSCs. Although the mean frequency of mEPSCs in cells expression Arc_(WT)_ appeared reduced, this was not significant as there was large variability between cells. ***H***, Representative average mEPSC waveforms recorded at a holding potential of −60 and + 40 mV for cells expressing GFP, Arc_(WT)_, and Arc_(W197A)_ in the presence of spermine (100 µm) in the intracellular solution. ***I***, Bar chart plotting the mean rectification index (peak amplitude at +40 mV divided by peak amplitude at −60 mV) for neurons expressing GFP (*n* = 9 cells; 0.34 ± 0.015), Arc_(WT)_ (*n* = 9 cells; 0.62 ± 0.016), and Arc_(W197A)_ (*n* = 6 cells; 0.45 ± 0.015). Thus, Arc_(WT)_ reduces the amount of rectification (as seen as an increase in the rectification index), whereas Arc_(W197A)_ has significantly less effect on rectification. Error bars in ***F***, ***G***, and ***I*** are SEM. ***p<0.001, **p<0.01. Statistical significance was tested using the Mann–Whitney test. Scale bar, 10 µm.

Previous findings have demonstrated that under basal conditions hippocampal cultured neurons overexpressing Arc-WT have significantly less AMPAR on their surface than neighboring untransfected neurons (Shepherd et al., 2006). There is also a significant reduction in the amplitude of AMPAR-mediated synaptic currents in CA1 neurons overexpressing Arc-WT protein in hippocampal slices (Rial Verde et al., 2006). Conversely, cultured hippocampal neurons from Arc knock-out mice exhibit an increased density of AMPAR at the cell surface and a deficit in AMPAR endocytosis (Chowdhury et al., 2006). Because Arc facilitates endocytosis of AMPAR and we have demonstrated that Arc directly binds to the AP-2 complex, we predicted that the Arc–AP-2 interaction regulates expression of synaptic AMPAR. To test our prediction, we first recorded AMPAR-mediated mEPSCs from cultured hippocampal neurons overexpressing an Arc-WT-GFP-tagged construct and from untransfected neighboring cells in the same cultures. This approach was used to negate any variations in AMPAR expression, which may arise from differences in cell density. Recordings from cells expressing EGFP alone were used as a control for transfection. In agreement with previous studies, a significant decrease in mEPSC amplitude was observed in cells overexpressing Arc_(WT)_ compared with untransfected neighboring cells (Fig. 4*Ai*; Rial Verde et al., 2006; Shepherd et al., 2006). Examination of the amplitude probability curves from Figure 4*a* shows that the majority of AMPAR-mediated mEPSCs had smaller amplitudes in the cell where Arc_(WT)_ was overexpressed (peak shifted to the left, red trace) compared with the untransfected neighboring cell (black trace). In contrast, there was no significant difference in the amplitude of mEPSCs recorded in an eGFP-expressing cell and its untransfected neighbor (Fig. 4*Bi*; Table 1).

To test whether the Arc-mediated reduction in the AMPAR mEPSC amplitude is dependent on an interaction with AP-2, we recorded mEPSCs from hippocampal cultures overexpressing either Arc_(195-199A)_- or Arc_(W197A)_- GFP-tagged mutant constructs. As predicted, the reduction in AMPAR-dependent mEPSC amplitudes observed in cells overexpressing Arc_(W197A)_ or Arc_(195-199A)_ was significantly less pronounced compared with cells overexpressing Arc_(WT)_ (Fig. 4*C*,*Di*).Pooled data are displayed as cumulative probability distributions (Fig. 4*E*) and as bar charts plotting the mean amplitude and interval (Fig. 4*F,G*; Table 1).

**Figure 5.**
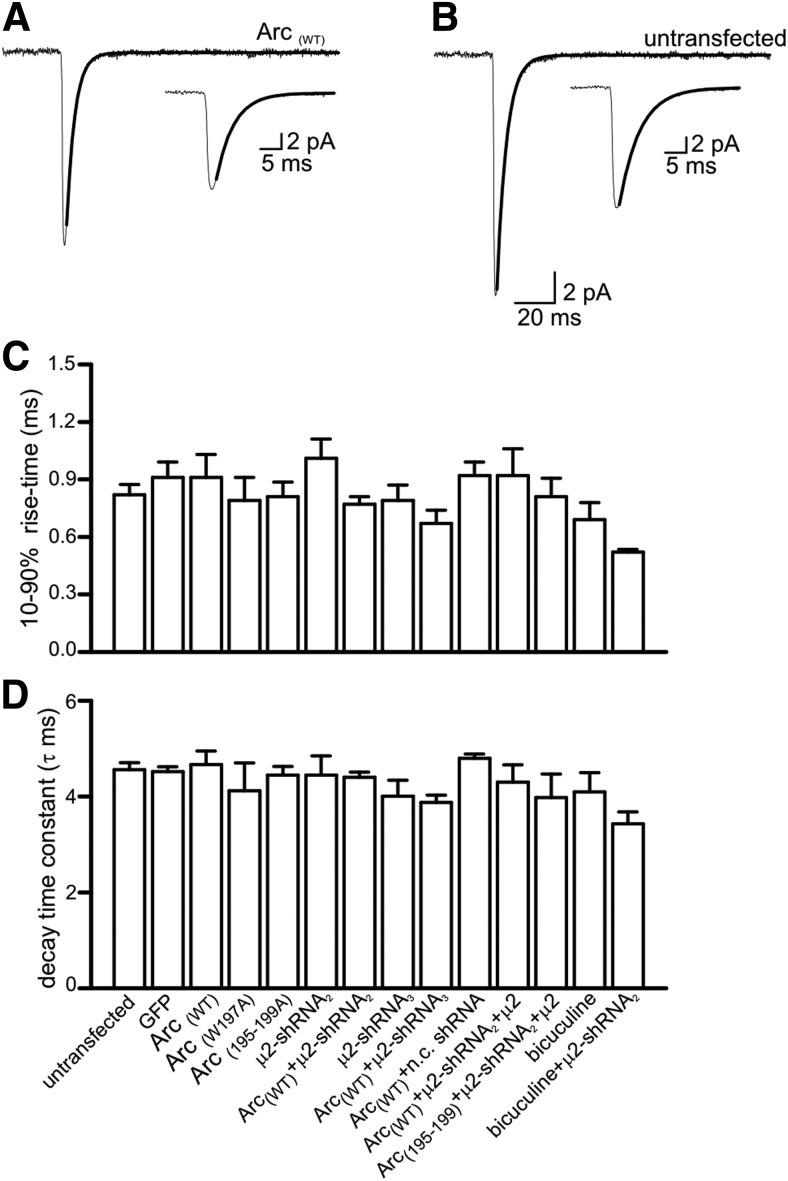
**Overexpression of Arc-cDNAs does not affect AMPAR-mediated mEPSC kinetics in hippocampal neurons. *A***, Average of 75 mEPSCs aligned on the midpoint of the rising phase) from an individual neuron expressing Arc_(WT)._ The decay was fitted with a single exponential (τ = 4.5 ms, black line). Inset, The average mEPSC at an expanded time base showing the exponential fit to the decay. ***B***, Average of 80 mEPSCs (aligned on the midpoint of the rising phase) from an untransfected neuron which was a close neighbor to the cell in ***A***. The decay of the mEPSC was very similar to the transfected neighbor (the decay was fitted with a single exponential; τ = 4.7 ms, black line). Inset, The average mEPSC at an expanded time-base to show the exponential fit to the decay. ***C***, Bar chart plotting the mean 10–90% rise time of mEPSCs recorded from untransfected neurons (*n*= 18) and from neurons expressing different constructs and in different conditions (*n* = 6 for each). The mean rise time was calculated by averaging the rise time of mean currents from individual recordings. There was no significant difference in the mean mEPSC rise time recorded from any of the neurons. ***D***, Bar chart plotting the mean decay time constant (τ) from untransfected neurons (*n*=18) and from neurons expressing different constructs and in different conditions (*n*=6 for each). The mean decay time constant (τ) was calculated by averaging the time constant from the decay of mean currents from individual recordings. The decay of mEPSCs was not significantly different between conditions. The error bars in ***C*** and ***D*** are SEM. Statistical significance was tested using the Mann–Whitney test

Our biochemical data show that Arc preferentially internalises GluA1 rather than GluA2 subunits (Fig. 3*A,B*). To test whether Arc has similar effects on the endogenous AMPA receptors, which are expressed at synapses, we measured the rectification of AMPA receptor mediated mEPSC amplitudes. The reduction in the surface expression of synaptic AMPA receptors containing GluA1 subunits would be expected to reduce rectification at positive holding potentials (Bowie and Mayer, 1995; Kamboj et al., 1995; Plant et al., 2006). As predicted, the rectification index (calculated by dividing the amplitude of mEPSCs at +40 mV by the amplitude at −60 mV) was significantly increased in cells expressing Arc_(WT)_ compared with GFP- and Arc_W197A_-expressing cells (Fig. 4*H,I*). Neither mEPSC rise or decay kinetics were significantly effected by overexpression of Arc_(WT)_, Arc mutants, or eGFP (Fig. 5*A*–*D*). The consistency in mEPSC rise and decay kinetics across recordings (Fig. 5*C*,*D*; Table 1) demonstrates that any changes in mEPSC amplitude are a result of receptor internalisation rather than variations in recording quality. These experiments suggest that the reduction in mEPSC amplitude induced by Arc_(WT)_ overexpression in hippocampal neurons is dependent on the binding of Arc_(WT)_ to the AP-2 complex.

### The AP2 subunit μ2 is required for the Arc_(WT)_-induced reduction in mEPSC amplitude

**Figure 6.**
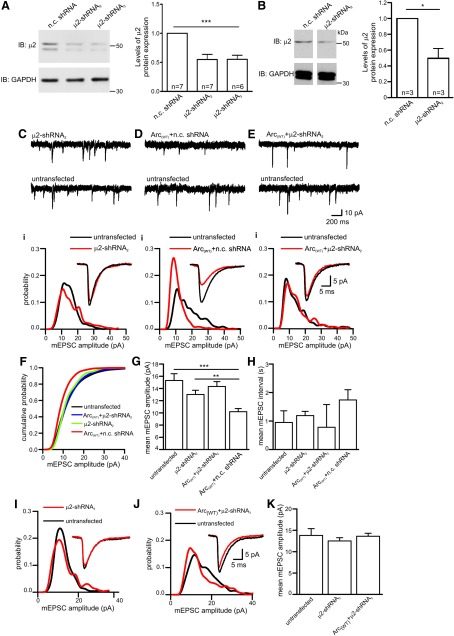
**AP-2 is required for Arc-dependent changes in synaptic strength. *A***, Blots showing levels of µ2 protein obtained from NSC cells overexpressing n.c. shRNA, µ2-shRNA_2,_ µ2-shRNA_3_ plasmids for 3–4 d. GAPDH was used as loading control. Bar chart plotting the analysis of µ2 band intensity normalized by GAPDH. Error bars indicate ± SEM and significance was tested using one-way ANOVA. ***p=0.0001. ***B***, Blots showing levels of µ2 protein obtained from cultured hippocampal neurons infected for 8–9 d with lentiviruses expressing either µ2-nc shRNA or µ2-shRNA_2_ sequences for 8–9 d. GAPDH was used as loading control. Bar chart plotting the analysis of µ2 band intensity normalized by GAPDH intensity. Error bars indicate ± SEM and significance was tested using one-way ANOVA. *p=0.019. ***C***, Representative AMPAR-mediated mEPSC traces from a neuron expressing µ2-shRNA_2_ and an untransfected neighbor. ***Ci***, Amplitude probability distributions from the neuron shown in ***C***. Note that reduction of AP-2 expression (µ2-shRNA_2_) has little effect on mEPSC amplitude. Inset, superimposed average mEPSC waveforms. ***D***, Representative AMPAR-mediated mEPSC traces from a neuron coexpressing Arc_(WT)_ and nc shRNA and an untransfected neighbor. ***Di***, Amplitude probability distribution from the neurons in ***D***. Note that coexpression of a n.c. shRNA does not prevent overexpression of Arc from reducing mEPSC amplitude. Inset, Superimposed average mEPSC waveforms. ***E***, Representative AMPAR-mediated mEPSC traces from a neuron coexpressing Arc_(WT)_ and µ2-shRNA_2_ and an untransfected neighbor. ***Ei***, Amplitude probability distribution from the neurons showed in ***E***. Note that coexpression of µ2-shRNA_2_ prevents the effects of Arc_(WT)_ on mEPSC amplitude. Inset, Superimposed average mEPSC waveforms. ***F***, Cumulative probability distributions for cells expressing shRNA_2_ (9 neurons), Arc_(WT)_ + shRNA_2_ (16 neurons), Arc_(WT)_+n.c shRNA (7 neurons), and for untransfected cells (12 neurons). ***G***, Bar chart plotting mean mEPSC amplitude for the cells in ***f***. Expression of shRNA_2_ prevented the Arc_(WT)_ overexpression effect of significantly reducing mEPSC amplitude (mean mEPSC amplitude 15.3 ± 1 pA in untransfected cells, Arc_(WT)_ + shRNA_2_ 14.3 ± 0.8 pA; *p*=0.52). Expression of shRNA_2_ alone had no significant effect on mEPSC amplitude (13 ± 0.7 pA; *p*=0.07), whereas Arc_(WT)_ + n.c. shRNA significantly reduced mEPSC amplitude (10.2 ± 0.53 pA; *p*=0.001). ***H***, Bar chart plotting the mean interval between mEPSCs for the cells in ***F***. The error bars in ***G*** and ***H*** are SEM. ***p<0.001, **p<0.005. Statistical significance was tested using the Mann–Whitney test. ***I***, Amplitude probability distributions for a neuron expressing µ2-shRNA_3_ and an untransfected neighbor and for a neuron overexpressing Arc_(WT)_ with µ2-shRNA_3_ and an untransfected neighbor (***J***). Inset, Superimposed average mEPSC waveforms. ***K***, Bar chart of mean mEPSC amplitudes for untransfected cells (*n* = 8), cells transfected with μ2-shRNA_3_ (*n*=10) and cells transfected with Arc_(WT)_+ μ2-shRNA_3_ (*n*=6). Neither expression of μ2-shRNA_3_ or Arc_(WT)_+μ2-shRNA_3_ significantly changed mEPSC amplitude (*p*=0.68 and *p*=0.27, respectively).

Previous studies have shown that depletion of the µ2 subunit compromises the stability of the remaining subunits of AP-2 and also that the complexes lacking the µ2 subunit are inactive and fail to localize to the plasma membrane (Meyer et al., 2000; Peden et al., 2002; Motley et al., 2003). To further investigate the importance of the Arc–AP-2 interaction, we designed shRNA-like sequences to knockdown the endogenous expression of µ2 in mouse tissue. We then used these shRNA constructs to transfect the mouse cell line NSC-34. A shRNA sequence, not predicted to knockdown any vertebrate genes, was used as a negative control. Using this approach, we identified two out of three shRNA sequences (µ2-shRNA_2_ and µ2-shRNA_3_) that efficiently reduced the protein expression of µ2 in NSC-34 cells (Fig. 6*A*). To knockdown endogenous µ2 in neurons, we generated lentiviruses expressing these two shRNAs. The lentiviruses also express emGFP isocistronically, to efficiently identify the transduced neurons. Lentiviral transduction of µ2-shRNA_2_ into hippocampal cultures resulted in an overall 50% reduction in µ2 expression compared with the negative control shRNA (Fig. 6*B*). Note that even under optimal circumstances transduction rates in primary neurons are between 70% and 80% using lentiviral systems. This indicates that a significantly more pronounced reduction in µ2 expression has been achieved in those cells that have been transduced and used for recordings. To examine whether AP-2 is required in AMPAR-mediated synaptic transmission under basal conditions, we first transduced hippocampal cultures at 6–7 DIV with a lentivirus expressing µ2-shRNA_2_-emGFP and recorded AMPAR-mediated mEPSCs 7–8 d after transfection. No significant change in mEPSC amplitude was observed in cells expressing µ2-shRNA_2_ alone compared with untransfected neighboring cells (Fig. 6*Ci*). These findings suggest that the constitutive endocytosis of AMPAR occurring under basal conditions in cultured hippocampal neurons is not strictly dependent on AP-2.

To test whether AP-2 is required for Arc-mediated endocytosis of AMPAR, we recorded mEPSCs from hippocampal neurons expressing either µ2-shRNA_2_-emGFP- plus mCherry-Arc-WT or the negative control (n.c.) shRNA-emGFP plus mCherry-Arc-WT, as well as untransfected neighboring neurons. Consistent with our hypothesis, a 30% reduction in mEPSC amplitudes was seen in neurons expressing Arc_(WT)_ plus n.c. shRNA (Fig. 6*Di*). However, this reduction in mEPSC amplitude was abolished in cells coexpressing Arc-WT plus µ2-shRNA_2_ (Fig. 6*Ei*). Pooled data are displayed as cumulative probability distributions (Fig. 6*F*) and as bar charts plotting the mean amplitude and interval (Fig. 6*G*,*H*; Table 1). To confirm this observation, we also recorded AMPAR-mediated mEPSCs from neurons expressing either µ2-shRNA_3_ alone or together with Arc-WT. Again, no change in mEPSC amplitudes was seen in cells expressing µ2-shRNA_3_ alone (Fig. 6*I*,*J*). However, expression of µ2-shRNA_3_ blocked the Arc-WT-mediated decrease in mEPSC amplitudes (Fig. 6*I*–*K*). Neither mEPSC rise or decay kinetics was significantly affected by overexpression of either µ2-shRNA_2_ or µ2-shRNA_3_ alone, Arc-WT plus µ2-shRNA_2_ or_,_ µ2-shRNA_3_, or Arc_(WT)_ plus n.c. shRNA (Fig. 5). These results demonstrate that knock-down of the AP-2 complex is sufficient to disrupt the Arc-mediated endocytosis of AMPAR. Together, these findings suggest that AP-2 is required for the Arc-mediated endocytosis of AMPAR in hippocampal neurons.

### Arc-mediated reduction in AMPAR-mediated mEPSC amplitude requires the binding of Arc to AP-2

**Figure 7.**
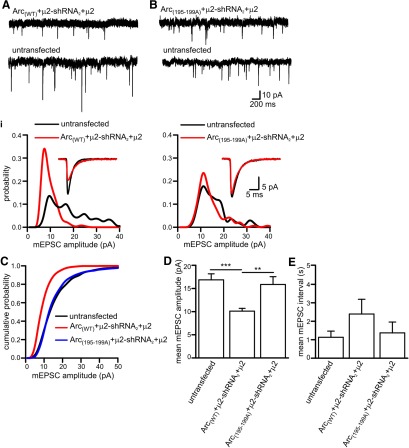
**Arc–AP-2µ interaction is required for the Arc-mediated reduction in AMPAR mEPSC amplitude. *A***, Representative AMPAR-mediated mEPSC traces from a neuron expressing Arc_(WT)_+µ2-shRNA_2_+µ2 and an untransfected neighboring neuron. ***Ai***, Amplitude probability distributions from the neurons showed in A. Note that reintroduction of µ2 rescued the effect of Arc_(WT)_ overexpression leading to a reduction in the amplitude of mEPSC amplitudes (shift to the left, red trace). Inset, Superimposed average mEPSC waveforms. ***B***, Representative AMPAR-mediated mEPSC traces from a neuron expressing Arc_(195-199A)_+µ2-shRNA_2_+µ2 and an untransfected neighboring neuron. ***Bi***, Amplitude probability distributions from the neurons showed in ***D***. Note that reintroduction of µ2 has little effect in mEPSC amplitude (no shifts between black and red traces). Inset, Superimposed average mEPSC waveforms. ***C***, Cumulative probability distributions for cells expressing Arc_(WT)_ +μ2-shRNA_2_ +μ2 (14 neurons), Arc_(195-199A)_ +μ2-shRNA_2_ +μ2 (9 neurons), and untransfected cells (14 neurons). ***D***, Bar chart plotting mean mEPSC amplitude for the cells in ***C***. Expression of μ2 rescued the reduction in mEPSC amplitude produced by Arc_(WT)_ overexpression, following the knockdown of AP-2 by shRNA_2_ (mean mEPSC amplitude in untransfected cells 16.9 ± 1.3 pA vs 10.1 ± 0.6 pA in cells expressing Arc_(WT)_ +μ2-shRNA_2_ +μ2; *p*=0.0001). In contrast, expression of μ2 had no significant effect on mEPSC amplitude when Arc_(195-199A)_, which does not interact with AP2, was expressed together with shRNA_2_ (15.9 ± 1.7 pA; *p* = 0.46). ***E***, Bar chart plotting the mean interval between mEPSCs for the cells in ***C***. The error bars in ***D*** and ***E*** are SEM. ***p<0.001, **p<0.01. Statistical significance was tested using the Mann–Whitney test.

We have shown that: (1) the reduction in AMPAR-mediated mEPSC amplitude observed in neurons overexpressing Arc_(WT)_ is either reduced or abolished in neurons expressing mutated Arc, which cannot bind to AP-2 (Fig. 4); and (2) that the effect of Arc-WT overexpression on mEPSC amplitude is reduced in neurons expressing a decreased amount of AP-2µ2 protein (Fig. 6). These data suggest that Arc requires AP-2 to facilitate the internalization of AMPAR. To confirm the functional relationship between Arc and AP-2, we recorded mEPSCs from hippocampal neurons expressing Arc-WT and µ2-shRNA_2_-emGFP in the same lentivirus combined with re-expression of µ2 using another lentivirus expressing a µ2-shRNA_2_ resistant µ2-mCherry fusion protein. As a control, lentiviruses encoding Arc_(195-199A)/_µ2-shRNA_2_-emGFP and µ2-mCherry was used to transduce hippocampal cultures. As predicted, the Arc_(WT)-_mediated reduction in AMPAR mEPSC amplitude caused by depletion of AP-2/µ2 (Fig. 4) was reversed by overexpressing µ2 (Fig. 7*Ai*), demonstrating that AP-2/µ2 is specifically required for the effect of Arc on AMPAR amplitudes. In contrast, no effect on AMPAR amplitudes was seen in cells expressing a mutant form of Arc_(195-199A)_ that cannot bind to AP-2, irrespective of the expression status of µ2 (Fig. 7*Bi*,*C*). Pooled data are displayed as cumulative probability distributions (Fig. 7*C*) and as bar charts plotting the mean amplitude and interval (Fig. 7*D*,*E*; Table 1). Neither mEPSC rise or decay kinetics were significantly affected by overexpression of Arc-WT-µ2-shRNA_2_-emGFP plus µ2-mCherry and Arc_(195-199A)_-µ2-shRNA_2_-emGFP plus µ2-mCherry (Fig. 5). These experiments clearly demonstrate that the Arc–AP-2 interaction is required for the reduction in AMPAR-mediated mEPSC amplitudes rather than sole disruption in AP-2.

### AP-2 is required for Arc-dependent homeostatic scaling

**Figure 8.**
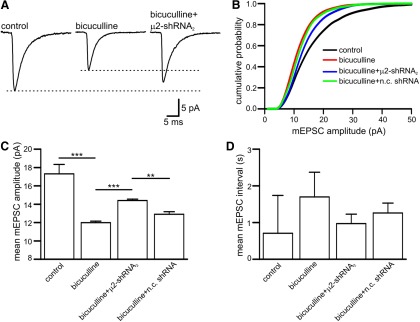
**AP-2 is required for Arc-dependent homeostatic scaling. *A***, Average mEPSC waveforms from an untransfected neuron cultured in control conditions, from an untransfected neuron exposed to bicuculline and from a µ2-shRNA_2_ expressing neuron that has been incubated in bicuculline (40 µm; 48 h). Note that the bicuculline-induced down regulation of the mEPSC amplitude was reduced in AP-2 depleted cells (µ2-shRNA_2_ expressing cells).The untransfected neuron and the neuron expressing µ2-shRNA_2_ that were cultured in the presence of bicuculline were neighbors in the same dish, while the untransfected neuron cultured in control conditions was from the same preparation. ***B***, Cumulative amplitude distribution for untransfected neurons cultured in control conditions (black line, *n*=10 neurons), untransfected neurons incubated in bicuculline (red line, *n*=15 neurons), µ2-shRNA_2_ expressing cells incubated in bicuculline (blue line; *n*=6 neurons) and cells transfected with n.c. shRNA incubated in bicuculline (green line; *n*=5 neurons). ***C***, Bar chart plotting the mean mEPSC amplitude for the cells shown in ***B***. Incubation in bicuculline significantly reduced the mean mEPSC amplitude (from 17.3 ± 1 pA to 11.9 ± 0.2 pA; *p*=0.0001). Expression of shRNA_2_ significantly increased mEPSC amplitude in bicuculline (14.38 ± 0.16 pA; *p*=0.0001), whereas n.c shRNA had significantly less effect (12.9 ± 0.28 pA; *p*=0.007). ***D***, Bar chart plotting the mean interval between mEPSCs for the cells in ***B*** and ***C***. The error bars in ***C*** and ***D*** are SEM. ***p<0.001, **p<0.01. Statistical significance was tested using the Mann–Whitney test.

Homeostatic scaling is the ability of neurons to sense the level of synaptic activity and compensate for changes by modulating their excitability. For example, in response to a prolonged increase in synaptic activity, neurons reduce synaptic strength by facilitating endocytosis of synaptic AMPAR (downscaling). Arc, whose expression is robustly induced by increased activity, is known to facilitate synaptic downscaling by enhancing AMPAR endocytosis (Shepherd et al., 2006; Mabb et al., 2014). Because we have shown that AP-2 is required for the Arc-dependent endocytosis of AMPAR, we hypothesized that a reduction in AP-2 expression should impair Arc-dependent synaptic scaling. To test this, we recorded AMPAR-mediated mEPSCs from hippocampal, cultured neurons chronically treated with bicuculline (40 µm, 48 h), which blocks inhibitory neurotransmission mediated by GABA_A_ receptors and thus increases neuronal firing. In agreement with previous studies (Shepherd et al., 2006; Mabb et al., 2014), we observed a significant decrease in the amplitude of AMPAR-dependent mEPSCs in cells incubated with bicuculline compared with control cells (Fig. 8*A*–*C*). To address whether AP-2 was required for this reduction in mEPSC amplitude, we reduced µ2 expression by transducing hippocampal neurons with µ2-shRNA_2,_ and as a control, n.c. shRNA, for 5 d prior to bicuculline incubation. In neurons expressing the n.c. shRNA, bicuculline incubation still resulted in a robust reduction in mEPSC amplitude (Fig. 8*B*,*C*). However, the reduction in mEPSC amplitude was significantly smaller in neurons expressing µ2-shRNA_2_ (Fig. 8*A*–*C*). None of the treatments significantly (*p*>0.05) changed the frequency of mEPSCs (Fig. 8*D*; Table 1) or the rise or decay kinetics of mEPSCs (Fig. 5). Together, these findings support the hypothesis that the Arc–AP-2 interaction is required for the endocytosis of AMPAR during homeostatic scaling.

## Discussion

The present study identifies a functional link between Arc and the AP-2 complex, a vital component of CME pathway. The AP-2 complex is required for selection and recruitment of the endocytic cargo and also for clathrin recruitment to the plasma membrane, processes that initiate the formation of the clathrin-coated pit (Robinson, 2004; Saheki and De Camilli, 2012; Kelly et al., 2014; Kirchhausen et al., 2014). Here, we demonstrate that Arc immunoprecipitates with the AP-2 complex in hippocampal lysate and that Arc directly binds to the AP-2 complex (Fig. 1). We also show that the Arc residues _195_QSWGP_199_ mediate the Arc–AP-2 association and that a conserved tryptophan residue at position 197 (W_197_) is essential for this interaction (Fig. 2). Importantly, the GST-Arc mutants that are impaired in AP-2 binding still bound to another binding partner, Triad3A, demonstrating the structural integrity of the mutated Arc proteins. Interestingly, the mutated Arc proteins pulled down higher levels of Triad3A compared with GST-Arc_(WT)_ from cell extracts (Fig. 2). Although the reasons for these results were not addressed here, one possible explanation is that preventing the AP-2 interaction may render Arc's C-terminal domain more accessible to make contacts with Triad3A, leading to increased binding. Importantly, this apparently higher affinity for the ubiquitin ligase Triad3A does not cause changes in the expression/stability of the Arc mutants (Fig. 3). This further demonstrates that the observed functional changes of the Arc mutants are specifically due to the loss of its binding to AP-2. In agreement with previous studies (Shepherd et al., 2006; Waung et al., 2008), we showed that overexpression of Arc strongly reduces surface expression of GluA1, but not GluA2 in H4 neuroglioma cells (Fig. 3). In cultured hippocampal neurons, overexpression of Arc reduces the number of synaptic AMPA receptors, as shown by a decrease in the amplitude of AMPAR-mediated mEPSCs and also regulates AMPA receptor subunit composition (Fig. 4). It was previously shown that AMPAR containing GluA2 subunits show a linear current–voltage relationship in contrast to GluA2-lacking receptors that show pronounced rectification (Isaac et al., 2007). In our experiments, mEPSCs recorded from neurons overexpressing GFP alone showed pronounced rectification, suggesting that the predominant combination of AMPA receptors lacks the GluA2 subunit (Eales et al., 2014). In contrast, overexpression of Arc resulted in diminished mEPSCs rectification, suggesting a reduction in the proportion of synaptic receptors that lack the GluA2 subunit. These findings are in agreement with previous studies showing that there is an increase in surface expression of GluA1, but not GluA2 subunits in hippocampal cultures obtained from Arc knock-out mice at non-stimulated conditions (Shepherd et al., 2006). Also knock-down of endogenous Arc in hippocampal cultures resulted in increased GluA1 subunits at the surface at non-stimulated conditions (Waung et al., 2008). Furthermore, application of DHPG (which induces an increase in Arc translation and protein expression) to cultured hippocampal neurons reduced rectification (Eales et al., 2014). As expected, mutation of the AP-2 binding site in Arc or depletion of AP-2µ2 compromises the capacity of Arc to reduce AMPAR-mediated mEPSC amplitudes (Figs. 4–6). Furthermore, the Arc-mediated reduction in AMPAR mEPSC amplitudes was rescued in cells where depletion of AP-2µ2 was reversed by reintroducing µ2 (Fig. 7). Importantly, this rescue was compromised in cells expressing a mutated form of Arc that cannot interact with AP-2 (Fig. 7). Furthermore, disruption of the Arc–AP-2 interaction by reducing the expression of AP-2µ2 also dampens the Arc-mediated reduction in synaptic strength observed in homeostatic synaptic downscaling (Fig. 8). Combined, these experiments demonstrate that Arc-dependent endocytosis of AMPARs requires an interaction of Arc with AP-2. It has been recently shown that dynamin activity is not required to reduce AMPA receptors surface levels induced by exposure to bicuculline and potassium chloride, suggesting that homeostatic downscaling may also be induced in a clathrin-independent manner (Glebov et al., 2015). Thus, we cannot discard the possibility that AMPA receptor endocytosis via an as yet non-identified clathrin/dynamin independent mechanism may contribute to regulate synaptic strength seen in homeostatic synaptic downscaling.

The requirement of Arc regulating synaptic plasticity in the hippocampus is well established (Rial Verde et al., 2006; Bramham et al., 2010; Jakkamsetti et al., 2013; Mabb et al., 2014). However, to utilize Arc as a potential therapeutic target, it would be beneficial to obtain its crystal structure. During the development of this project, no information on Arc structure was available. As we have discovered that the interaction between Arc and AP-2 depends on a short motif in the Arc sequence (195–199), we have undertaken homology modelling studies using the iTASSER suite (http://zhanglab.ccmb.med.umich.edu/I-TASSER; Roy et al., 2010) to investigate the structural properties of this region and to obtain clues as to the structural nature of the interaction interface. Unfortunately, we were not able to obtain a model with a reasonable confidence score. The main reason for this is that there are no other protein structures in the databank that are sufficiently related to Arc to allow modelling by homology approaches. Our attempts are in agreement with a recent study (Myrum et al., 2015) that also obtained models with low scores that were deemed to be only moderately reliable. In addition, the central region of the protein was suggested to be largely unstructured and flexible and the area containing the AP-2 interaction motif described in this study was not included in the models. Interestingly, another recent study (Zhang et al., 2015) has succeeded in obtaining a partial crystal structure of Arc, demonstrating that the C-terminal part of Arc is evolutionary similar to the Ty3/Gypsy retrotransposon and that it shows similarity to the HIV gag protein. However, the crystal obtained does not include the N-terminal sequences up to amino acid 206 and therefore does not include the AP-2 binding motif. Nevertheless, as both studies (and our own modelling approach) suggested that the AP-2 binding motif is in a flexible and at least partly unstructured region of the protein, it is highly likely that this region of Arc is able to serve as a binding platform for multiple partners, including AP-2.

**Figure 9.**
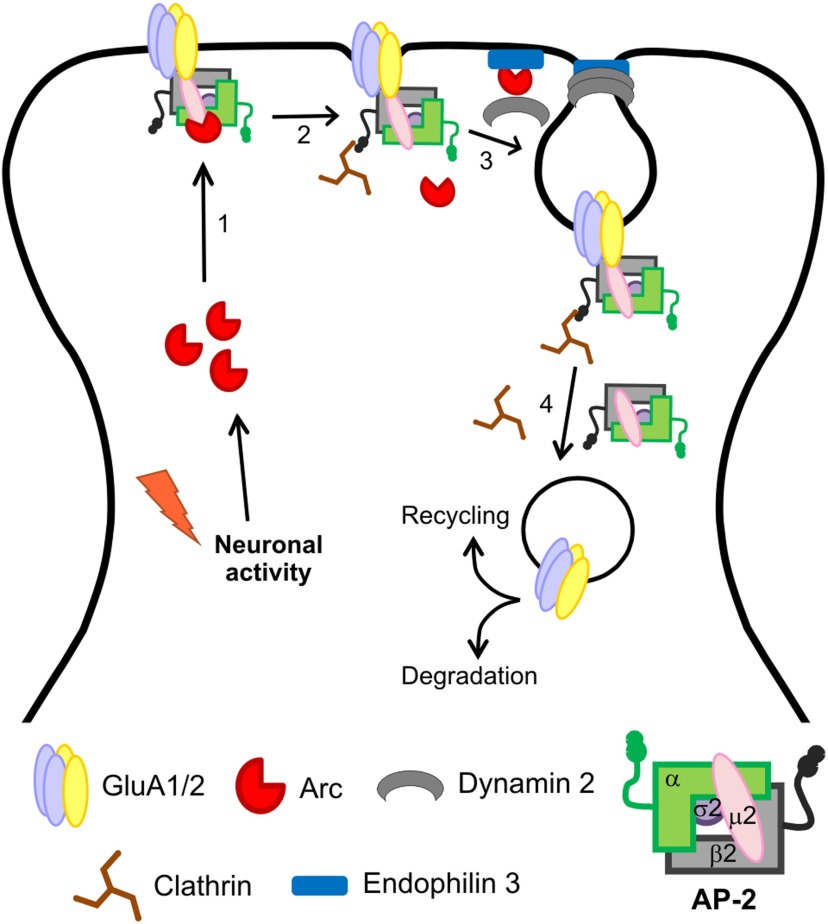
**Arc–AP-2 interaction controls synaptic strength**. The proposed model showing the mechanism by which Arc–AP-2 interaction facilitates AMPAR endocytosis. An increase in neuronal activity promotes rapid Arc mRNA translation and protein expression at the dendritic spines. ***1***, Newly expressed Arc binds to the AP-2 complex and may activate/facilitate AP-2 interaction with AMPAR at the plasma membrane. ***2***, To initiate the formation of the clathrin-coated assembly AP-2 binds and recruits clathrin to the membrane. ***3***, ***4***, Arc then binds and recruits endophilin and dynamin to promote scission of the endocytic vesicle containing the AMPAR to be targeted for either recycling or degradation.

Arc has been shown to mediate endocytosis of AMPAR via interaction with dynamin 2 and endophilin 3, which are accessory proteins of the CME machinery (Chowdhury et al., 2006). Endophilin and dynamin are required for membrane constriction and scission of the CCV, which are late events in the CME process. Recent evidence, using mature cultured cortical neurons from distinct knock-out mice where specific endophilins have been knocked out, clearly demonstrated that the assembly and early maturation events of clathrin-coated pit formation are independent of endophilin (Milosevic et al., 2011). Dynamin is recruited at late stages of endocytosis and its enrichment coincides with neck fission and release of the vesicle (Ferguson and De Camilli, 2012; Grassart et al., 2014). These findings clearly demonstrate that endophilin and dynamin do not participate in the cargo selection process. In contrast, AP-2 plays a critical role in the initiation of clathrin-mediated endocytosis, as it coordinates the cargo recruitment and selection together with clathrin recruitment and lattice assembly (Robinson, 2004; Kelly et al., 2014; Kirchhausen et al., 2014). The AP-2 complex is thought to exist in an inactive “closed” conformation in the cytosol that prevents unproductive interaction with clathrin. Binding to plasma membrane enriched phosphatidylinositol-4,5-bisphosphate [PtdIns(4,5)P2] and to transmembrane cargo, triggers conformational changes in AP-2 that are necessary to allow efficient binding to clathrin and bud formation which is thought to be the dominant mechanism for the initiation of clathrin coat assembly (Kelly et al., 2014). The current model, in which Arc is able to induce clathrin-mediated AMPAR endocytosis via interaction with endophilin and dynamin, does not place Arc as a decisive player in specifically controlling excitatory synaptic transmission. Importantly, our finding that Arc directly binds to AP-2 provides the mechanistic link by which activity-dependent expression of Arc specifically facilitates endocytosis of AMPAR. We therefore suggest a refined model where neuronal excitability induces an increase in Arc protein expression in dendritic spines (Fig. 9). Newly expressed Arc then interacts with AP-2 and possibly increases its affinity for the cytosolic tail of AMPA receptors. Activated AP-2 initiates the formation of the clathrin-mediated pits (CMPs) by coordinating the assembly of clathrin and binding to AMPAR at the postsynaptic density. We speculate that following the formation of CMP, Arc then binds and recruits endophilin and dynamin, which trigger fission of the vesicle neck. Arc may not be able to simultaneously bind to AP-2 and endophilin/dynamin. Therefore, one possible explanation is that following CMP formation, the affinity between Arc and AP-2 is reduced, releasing Arc from the CMP. The unbound Arc then binds and recruits endophilin and dynamin, which promotes neck fission and release of the CCV. Alternatively, Arc binding to dynamin/endophilin may be facilitated through AP-2 interacting partners, such as amphiphysin, which is able to bind to both AP-2 and dynamin (Slepnev et al., 2000). In fact, AP-2 has been described as a major hub for recruitment of accessory proteins to the maturing CMP (Schmid et al., 2006; McMahon and Boucrot, 2011). Our discovery that Arc directly binds to AP-2, which in turn regulates AMPAR endocytosis, provides the crucial mechanistic link explaining how activity-dependent expression of Arc regulates synaptic plasticity and therefore plays a critical role in learning and memory formation.

## Synthesis

The decision was a result of the Reviewing Editor Vivian Budnik and the peer reviewers coming together and discussing their recommendations until a consensus was reached. A fact-based synthesis statement explaining their decision and outlining what is needed to prepare a revision is listed below. The following reviewer(s) agreed to reveal their identity: Ursula Wyneken

Both reviewers agree that the study provides important advances to the field, as the work supplies evidence for a new mechanism that might explain aspects of the activity-dependent regulation of AMPAR trafficking at postsynaptic sites. While the observations are important, there are some issues that need to be resolved before the manuscript becomes suitable for publication in eNeuro. These issues, which should be addressed in a resubmission within 2 months, are outlined below. In addition, there are a number of comments by reviewers that might be helpful to authors to improve the manuscript, and that the authors might choose to address. These are outlined in the verbatim comments by reviewers, which are also enclosed.

1. It seems that there is a replication of some panels in the figures and the legends do not match with what is shown in the figures. This issue needs to be addressed before reviewers can properly judge the data.

2. Data should be provided as to which AP2 subunit binds Arc and the rationale for focusing on µ2.

3. The rationale and shortcomings of using miRNA vs siRNA to downregulate AP2 subunits should be discussed, as the specificity of this approach is questionable.

4. Evidence should be provided that the Arc alanine mutations specifically impair Arc's ability to interact with AP2 (as opposed to other Arc binding partners described in this or previous reports), and that these mutations do not alter the subcellular localization of Arc.

5. There should be a demonstration that the conclusion about the facilitation of AP2-induced GluA1 but not GluA2 receptor endocytosis is not due to overexpression of the receptor subunit types, and discuss the discrepancy with previously published studies, showing removal of the GluA2 subunit during Arc-dependent homeostatic plasticity. Further, electrophysiological recordings, substantiating that GluA1, but not GluA2 are preferentially internalized, should be provided.

6. To properly interpret and consolidate the electrophysiological and biochemical observations, the authors need to show that AP2 fails to bind Arc 195-199A.

7. The authors should avoid over-speculation in their model, or at least make clear which parts of their model are based on actual data in this manuscript and which parts are purely speculative.

8. It should be feasible to readily address other minor comments detailed by reviewers.

Reviewer's comments

Reviewer 1

The work provides advances in the field because it provides a new mechanistic explanation for activity-dependent AMPAR internalization. Specifically, a direct interaction between the activity-regulated cuyoskeleton associated protein Arc/Arg3.1 and AP-2 (a clathrin-adaptor protein) is shown. This interaction might be necessary for GluA1 subunit endocytosis and is proposed to be relevant in homeostatic and constitutive AMPAR recycling. The experimental approach regarding the first part of the hypothesis (i.e. interaction of Arc with AP-2 complex) is correct.

However, the present findings are opposite to other recently published findings and this should be discussed properly. Similarly, the relevance of the present finding in homeostatic plasticity remains to be fully established and this requires an adequate discussion.

The abstract clearly summarizes the work and provides a clear message regarding a novel mechanism involved in Arc-dependent AMPAR internalization.

Comments:

The authors describe that interaction of the activity-regulated and cytoskeleton associated protein Arc with the clathrin adaptor protein complex AP-2 leads to AMPAR internalization, affecting putatively selectively GluA1 subunits. The interaction of Arc with the AP-2 complex is well-demonstrated. Moreover, the primary amino-acid sequence within Arc (and within it, a single W residue)that mediates this interaction was now identified. This is an interesting finding. However, several issues are not clear:

•Which of the AP-2 subunits is involved in this interaction? This is important because later, experiments are focused on µ2

•Why authors use miRNA instead of siRNA to target µ2 subunits? Which other genes are targets of this miRNA sequence? One miRNA may target over hundred genes and this is not discussed. Is it possible that the rescue experiments by over-expressing µ2 subunits is due to a stimulatory effect of µ2 per se on AMPAR internalization? A control experiment of the sole effect of µ2 overexpression is required! (Fig. 7)

•Although in the introduction the relevance of the proposed mechanism for homeostatic scaling is posed, this is not further addressed in the discussion. Moreover, relevant papers in the field are not discussed. The present findings need to be discussed in light of recent findings of homeostatic AMPAR downscaling that is clathrin-independent (Glebov et al (2014) J Neuroscience 35:483; Qin et al (2015) JBC; and with opposing findings of Rial Verde (2006) showing a Arc-induced reduction of GluA2-containing AMPARs.

•Finally, no efforts were undertaken in electrophysiological recordings to confirm that GluA1, but not GluA2, are preferentially internalized in the present experimental conditions. This can be easily done e.g. by analyzing inward rectification properties of AMPARs.

•Methotodological points (minor):

1)The centrifugal force in centrifugation steps needs to be expressed as g, and not rpm (the centrifugal force varies with the radius of the rotor). Please correct.

2)The composition of the lysis buffer in immunoprecipitation experiments is not mentioned.

3)The amplitude of representative tracings in the electrophysiological recordings are not convincing (Figs. 4, 6, 7), because average tracings (e.g. 4i, inset) are similar in amplitude to the maximal (but not average) amplitude of the corresponding mEPSC trace (e.g. 4D). Revise scale bars.

4) The statistical test is Tukey (not Turkey), please correct

Reviewer 2

It has been reported that Arc protein is involved in AMPA receptor internalization. However, the Arc-mediated internalization mechanism is still speculative. In this manuscript, the authors reported the direct association of Arc and clathrin-adaptor protein 2 (AP-2), the essential component of clathrin-mediated endocytosis. This is the first report proposing that Arc regulates receptor internalization through the direct binding with endocytic machinery.

I think that this manuscript has significant impact on elucidating the molecular mechanisms of glutamate receptor trafficking and will contribute for understanding the molecular basis of neuronal homeostasis.

Arc is one of the major regulators that determines the number of AMPA receptors at postsynaptic sites in a neuronal activity-dependent manner. Authors presented evidences supporting that Arc induces AMPAR internalization through the direct binding with clathrin-adaptor protein 2 (AP-2), the core component of clathrin-mediated endocytosis. Their biochemical and electrophysiological results may give the significant impact on understanding the molecular mechanisms of Arc-mediated AMPAR internalization. However, the following concerns are still remained to be addressed, and extensive revision of the manuscript is necessary.

Major comments

1.Exact same figures have been used in Figure 4A-G and Figure 6C-I, and Figure 6 legend does not match with the actual figure. Any comments and discussion related to this section will be made once authors address this issue.

2.The authors have performed mass spectrometric analysis against Arc-immuno-precipitated proteins prepared from mice hippocampal lysate. They have identified AP-2, dynamin1, beta-CaMKII, PSD95 as the potential Arc binding partners. Further biochemical analysis confirmed that AP-2 protein complex directly bind to Arc through 195QSWGP199 motif in the middle of Arc (Figure 1 and 2). Prompted by the structural analysis, the authors performed electrophysiological experiments by using two Arc alanine mutants (ArcW197A and Arc_195-199A mutants) in Figure 4-7, and concluded that the specific protein interaction between Arc and AP-2 is critical for Arc-mediated down scaling of synaptic transmission.

However, the authors can conclude the interaction only when these alanine mutants specifically lack the interaction with AP-2 and have comparable subcellular distribution pattern compared with Arc(WT). It is possible that alanine mutations in the middle of Arc may lead to the gross conformation change of the protein that may cause the change of protein-protein interactions and pattern of subcellular localization of Arc mutants.

The authors found by mass spec analysis, dynamin1, beta-CamKII and PSD95 are in Arc protein complex. In addition, it has been described that Arc binds to other synaptic molecules, including TARP, WAVE1, GKAP, GluN2A and GluN2B (Zhang W., et al., 2015, Neuron, 86, 490-500). Many of these Arc binding proteins play critical roles in synaptic transmission and plasticity, and the lack of the interaction between other synaptic protein(s) and Arc alanine mutants may cause the reduced AMPAR synaptic transmission. In addition, abnormal subcellular targeting of Arc by alanine mutations may cause the lack of down scaling of AMPAR mEPSC. Therefore, authors should provide evidence showing that the Arc alanine mutants specifically lost the binding ability with AP-2 without changing the pattern of subcellular localization.

3.From Fig3's experiments, authors claimed that Arc facilitates AP2-induced GluA1 endocytosis but not GluA2. Do the overexpressed myc-GluA1 or myc-GluA2 in this experiment form homertetramer or heterotetramer? Is this selectivity observed in endogenous GluA1 or GluA2 endocytosis? How do the authors consolidate their findings of this selectivity towards GluA1 with previous evidence of a selective removal of GluA2 containing AMPAR during arc-dependent homeostatic scale down?

4.The reagents tested in biochemistry and electrophysiology should be the same. The authors used Arc_195-199A mutant in electrophysiology without presenting biochemical characterization in Figure 2. The authors need to present the IP data showing the lack of AP-2 binding with Arc_195-199A mutant.

Minor comments

1.The blotting patterns of GAPDH in Fig. 6B is different from others (Figure 3A, 3B and 6A). The authors should confirm the antibody used for this experiment.

2.Figure 9 present the model how Arc-AP-2 binding regulates homeostatic downscaling. However, it is not appropriate to present this model at this stage of studies, since the most of the steps are based only on speculation, and not dependent on their results.

3.Fig1.G right. Change "Arc FL" label to "Arc(WT)" to be consistent with previous text.
